# Improvement of ethanol and 2,3-butanediol production in *Saccharomyces cerevisiae* by ATP wasting

**DOI:** 10.1186/s12934-023-02221-z

**Published:** 2023-10-08

**Authors:** Futa Yatabe, Taisuke Seike, Nobuyuki Okahashi, Jun Ishii, Fumio Matsuda

**Affiliations:** 1 Department of Bioinformatics Engineering, Graduate School of Information Science and Technology, Osaka University, 1-5 Yamadaoka, Suita, Osaka 565-0871 Japan; 2 Industrial Biotechnology Initiative Division, Institute for Open and Transdisciplinary Research Initiatives, Osaka University, 2-1 Yamadaoka, Suita, Osaka 565-0871 Japan; 3 Analytical Innovation Research Laboratory, Graduate School of Engineering, Osaka University Shimadzu, Osaka University, 2-1 Yamadaoka, Suita, Osaka 565-0871 Japan; 4 Engineering Biology Research Center, Kobe University, 1-1 Rokkodai, Nada, Kobe, Hyogo 657-8501 Japan; 5 Graduate School of Science, Technology and Innovation, Kobe University, 1-1 Rokkodai, Nada, Kobe, Hyogo 657-8501 Japan

## Abstract

**Background:**

“ATP wasting” has been observed in ^13^C metabolic flux analyses of *Saccharomyces cerevisiae*, a yeast strain commonly used to produce ethanol. Some strains of *S. cerevisiae*, such as the sake strain Kyokai 7, consume approximately two-fold as much ATP as laboratory strains. Increased ATP consumption may be linked to the production of ethanol, which helps regenerate ATP.

**Results:**

This study was conducted to enhance ethanol and 2,3-butanediol (2,3-BDO) production in the *S. cerevisiae* strains, ethanol-producing strain BY318 and 2,3-BDO-producing strain YHI030, by expressing the fructose-1,6-bisphosphatase (FBPase) and ATP synthase (ATPase) genes to induce ATP dissipation. The introduction of a futile cycle for ATP consumption in the pathway was achieved by expressing various FBPase and ATPase genes from *Escherichia coli* and *S. cerevisiae* in the yeast strains. The production of ethanol and 2,3-BDO was evaluated using high-performance liquid chromatography and gas chromatography, and fermentation tests were performed on synthetic media under aerobic conditions in batch culture. The results showed that in the BY318-opt_ecoFBPase (expressing opt_ecoFBPase) and BY318-ATPase (expressing ATPase) strains, specific glucose consumption was increased by 30% and 42%, respectively, and the ethanol production rate was increased by 24% and 45%, respectively. In contrast, the YHI030-opt_ecoFBPase (expressing opt_ecoFBPase) and YHI030-ATPase (expressing ATPase) strains showed increased 2,3-BDO yields of 26% and 18%, respectively, and the specific production rate of 2,3-BDO was increased by 36%. Metabolomic analysis confirmed the introduction of the futile cycle.

**Conclusion:**

ATP wasting may be an effective strategy for improving the fermentative biosynthetic capacity of *S. cerevisiae,* and increased ATP consumption may be a useful tool in some alcohol-producing strains.

**Supplementary Information:**

The online version contains supplementary material available at 10.1186/s12934-023-02221-z.

## Background

The exhaustion of petroleum reserves has recently stimulated advancements in biorefinery-linked technologies for generating fuels and chemicals from biomass, which is a renewable resource [1]. Bioethanol can be used as a substitute for petroleum-based transportation fuels and shows lower net emissions of the greenhouse gas CO_2_ [2]. The budding yeast *Saccharomyces cerevisiae* exhibits a high ethanol production capacity and is a representative host for bioethanol production [3–5]. *Saccharomyces cerevisiae* is a safe non-pathogenic organism and is extensively employed to synthesize various biochemicals and biofuels because of its tolerance to alcohol and harsh environments [6, 7]. Hence, the synthesis of higher alcohols (*n*-butanol [8] and 1,3-propanediol [9]) has progressed by utilizing *S. cerevisiae* as a host. Specifically, 2,3-butanediol (2,3-BDO) is a versatile chemical used to produce a diverse array of chemical products and is a crucial divalent alcohol applied as a raw material for pharmaceutical and cosmetic intermediates, inks, perfumes, liquid crystals, insecticides, and antifreezes [10]. Additionally, dehydration of 2,3-BDO generates 1,3-butadiene, a precursor for synthetic rubber, and methyl ethyl ketone, a liquid fuel additive [11, 12]. Therefore, investigations of 2,3-BDO synthesis using yeast have gained attention [13–15].

Although 2,3-BDO production by *S. cerevisiae* has several advantages, it also presents potential limitations. First, the expression of 2,3-BDO biosynthetic enzymes is low in wild-type *S. cerevisiae* strains, resulting in low 2,3-BDO production, typically around 100 mg/L [12]. This is because of the low intrinsic activities of acetolactate synthase (ALS) and butanediol dehydrogenase (BDH). Second, redox imbalances and other factors can lead to low yields and productivity of 2,3-BDO [12]. To overcome these limitations, we previously investigated the impact of heterologous overexpression of codon-optimized ALS from *Lactobacillus plantarum* and codon-optimized acetolactate decarboxylase (ALDC) from *Lactococcus lactis* [15]. Our resultant *S. cerevisiae* strain, YHI030, lost the ability to produce ethanol and acquired the ability to produce 2,3-BDO, achieving 81.0 ± 1.3 g/L 2,3-BDO production using 300 g/L glucose as a carbon source. This yield is comparable to the highest yield reported for any genetically engineered *S. cerevisiae* strain to date. However, YHI030 has a slow glucose consumption rate (504 h), highlighting the need to improve the 2,3-BDO production rate.

In yeast, ATP production and ethanol synthesis are bidirectionally coupled, and reductions in ATP levels must be counteracted by increased ATP synthesis via ethanol production. To enhance the alcohol production capacity of *S. cerevisiae* strains, reducing ATP production or increasing ATP turnover can help reduce ATP waste. We recently performed a metabolic analysis of the laboratory strain BY4947 and three industrial strains with high alcohol fermentation capacities, namely QA23, RedStar, and Kyokai 7, and determined the ATP regeneration and consumption rate based on the total metabolic flux distribution [16]. Our findings indicated that strains with higher specific growth rates have higher ATP consumption rates and that the rate of ethanol-specific production and the rate of ATP regeneration and consumption are positively correlated [16]. ATP consumption promotes ADP regeneration via substrate-level phosphorylation in glycolysis, which increases the rate of ethanol production because of overflow metabolism, or the ‘Crabtree effect.’ In addition, the rate of glucose-specific consumption is positively correlated with the rate of ATP regeneration and consumption [16]. Previous studies reported that increased ATP consumption in *Escherichia coli* and *S. cerevisiae* enhances substrate uptake and material production rates [17–20]. Although we have estimated the net ATP regeneration rate using ^13^C-based metabolic flux analysis, the purpose of ATP consumption by industrial strains remains unclear. Industrial strains tend to consume more ATP, even at higher growth rates, suggesting that ATP consumption is associated with ethanol production [16]. Therefore, we hypothesized that artificially consuming ATP could improve alcohol production capacity.

Various strategies have been employed to reduce ATP production and increase the ethanol fermentation capacity of yeast. One such approach is to replace the Embden-Meyerhof-Parnas pathway, which produces two moles of ATP per mole of glucose, with the Entner-Doudoroff pathway, which produces only one mole of ATP. However, attempts to introduce this pathway in *S. cerevisiae* by expressing the corresponding *E. coli* gene have been unsuccessful because of insufficient enzyme activity [21, 22]. Partial success has been achieved using sugars other than glucose or using sugar transporters to consume more ATP [23–25]. Another strategy to achieve “ATP wasting” is to introduce futile cycles, which are reaction cycles resulting in net energy waste. For example, in *E. coli*, a futile cycle involving phospho*enol *pyruvate synthase and pyruvate kinase increased the specific productivity of lactate by 25% [26]. In *S. cerevisiae*, a futile cycle based on the expression of pyruvate carboxylase and phospho*enol* pyruvate carboxykinase genes increased ethanol production per unit biomass [27].

In this study, we introduced several fructose-1,6-bisphosphatases (FBPases) and ATPase-based futile cycles into an *S. cerevisiae* S288C-derived ethanol-producing strain (BY318) and a 2,3-BDO-producing strain (YHI030) to increase the bioalcohol production rate.

## Results

### Introduction of a futile cycle into a laboratory strain (BY318)

**Fig. 1:**
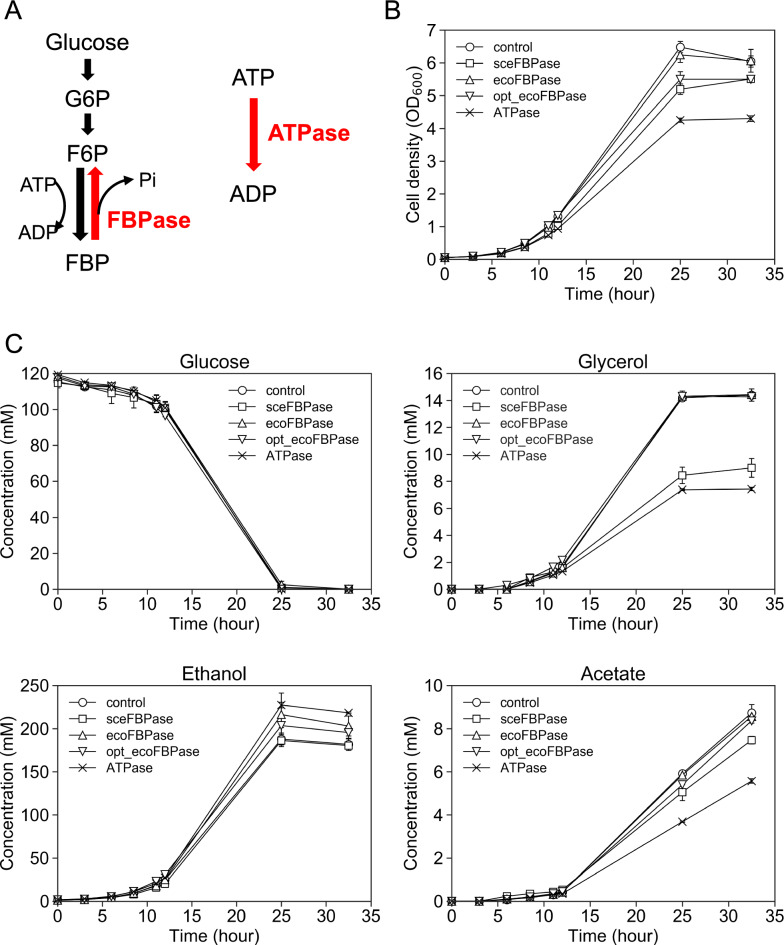
Fermentation profiles of BY318 strains in flask-scale batch cultivation using synthetic medium **A** Illustration of futile cycle using FBPase and ATPase. Time-course data of **B** cell density (OD_600_) and **C** concentrations (mM) of glucose, glycerol, ethanol, and acetate. BY318 strains were cultured in 50 mL of medium in a 200 mL baffled flask shaken at 120 rpm. Data are expressed as the mean ± SD (n = 3)

Our first target was to enhance the biosynthetic capacity of a laboratory strain (BY318) of *S. cerevisiae* by introducing an ATP-consuming cycle, or futile cycle. We sought to increase the growth rate, substrate consumption rate, and bioalcohol production rate of the strains. We introduced the fructose-1,6-bisphosphatase gene (*FBP1*) from *S. cerevisiae* (sceFBPase), *fbp* from *E. coli* (ecoFBPase), and a codon-optimized version of ecoFBPase for yeast (opt_ecoFBPase). FBPase is an enzyme present in many eukaryotes and is responsible for catalyzing the conversion of fructose 1,6-bisphosphate (FBP) to fructose 6-phosphate (F6P) and phosphate (Fig. 1A). Because the net reaction is ATP- > ADP + Pi when F6P + ATP- > FBP + ADP and FBP- > F6P + Pi occur in the glycolytic direction, artificial expression of FBPase may be used to establish this ATP-consuming reaction or futile cycle. However, as *S. cerevisiae* promotes glycolysis, FBPase is less active [28]. Hence, we synthesized and introduced the opt_ecoFBPase gene, which was codon-optimized for *S. cerevisiae*, to overcome potential translation rate-limiting issues associated with the heterologously expressed enzyme ecoFBPase from *E. coli*.

We also selected the F-ATPase (also referred to as ATPase) from *E. coli* for hydrolysis of ATP without perturbing the metabolites. The hydrophilic moiety of F-ATPase, F1-ATPase, is responsible for ATP hydrolysis or ADP phosphorylation [29] (Fig. 1A). In the absence of a proton gradient, ATP is simply hydrolyzed, which is performed by the αβγ subunit. To enable the simultaneous expression of these subunits in *S. cerevisiae*, we employed the 2A sequence-mediated linkage method, which allows for the co-expression of polycistronic genes in eukaryotes. Specifically, we used 2A sequences of GSG sequence + equine rhinitis B virus (ERBV-1) between *atpA* (α subunit gene) and *atpG* (β subunit gene) and GSG sequence + porcine teschovirus (PTV)-1 between *atpG* and *atpD* (γ subunit gene). ERBV-1 and PTV are highly peptide-cleavable 2A sequences in *S. cerevisiae*, and the addition of a GSG sequence at the beginning of these sequences improves their cleavage efficiency [30, 31]. We constructed strains in which the expression of the FBPases and ATPase genes was induced under the *PGK1* promoter, as described in the Methods section.

### Effect on growth and ethanol fermentation of laboratory strains introduced with a futile cycle

Batch cultures of five strains, including the control strain, were conducted in synthetic dextrose (SD) medium. Samples of the medium were collected at 0, 3, 6, 8.5, 11, 12, 25, and 32.5 h to monitor cell growth and changes in the medium composition over time. Growth was monitored by measuring the optical density at 600 nm (OD_600_), and extracellular metabolites were analyzed using high-performance liquid chromatography (HPLC) (Fig. 1). The BY318-ecoFBPase strain did not exhibit a significant difference in the maximum OD_600_ at 25 h compared to that of the BY318-Control strain, whereas the maximum OD_600_ of the other strains was decreased. Specifically, the BY318-sceFBPase, BY318-opt_ecoFBPase, and BY318-ATPase strains exhibited significantly lower maximum OD_600_ values, which were 20%, 15%, and 34%, respectively, lower than that of the BY318-Control strain (Fig. 1B). This may be because of the reduced biomass yield caused by ATP consumption. The specific growth rates of the BY318-sceFBPase, BY318-opt_ecoFBPase, and BY318-ATPase strains, determined from the approximate curves of growth behavior during the logarithmic growth phase (Table 1), were found to be 0.282 ± 0.005 h^−1^, 0.302 ± 0.000 h^−1^, and 0.275 ± 0.000 h^−1^, respectively.

**Table 1:** Growth rate and metabolite consumption and production rates and yields of BY318 strains

	Specific growth rate (h^−1^)	Specific glucose consumption rate (mmol/g-DCW/h)	Specific glycerol production rate (mmol (mmol/g-DCW/h)	Specific ethanol production rate (mmol (mmol/g-DCW/h)	Specific acetate production rate (mmol (mmol/g-DCW/h)	Glycerol yield (mol (mol-glucose)^−1^)	Ethanol yield (mol (mol-glucose)^−1^)	Acetate yield (mol (mol-glucose)^−1^)
control	0.304 ± 0.000	8.05 ± 0.42	1.11 ± 0.03	14.0 ± 0.4	0.242 ± 0.019	0.138 ± 0.006	1.75 ± 0.09	0.030 ± 0.002
sceFBPase	0.282 ± 0.005**	9.10 ± 1.55	1.30 ± 0.14	14.4 ± 1.2	0.351 ± 0.017**	0.145 ± 0.019	1.60 ± 0.17	0.039 ± 0.005*
ecoFBPase	0.302 ± 0.005	8.29 ± 1.55	1.08 ± 0.02	14.6 ± 1.4	0.247 ± 0.027	0.133 ± 0.022	1.78 ± 0.15	0.031 ± 0.008
opt_ecoFBPase	0.302 ± 0.000**	10.5 ± 0.3**	1.31 ± 0.02***	17.4 ± 0.6**	0.266 ± 0.008	0.125 ± 0.002*	1.66 ± 0.03	0.025 ± 0.001**
ATPase	0.275 ± 0.000***	11.4 ± 1.0**	1.19 ± 0.00**	20.3 ± 0.5***	0.286 ± 0.013*	0.105 ± 0.009**	1.79 ± 0.11	0.025 ± 0.001**

Asterisks indicate significant differences by two-tailed Student’s *t*-test, versus control. *p < 0.05, **p < 0.01, ***p < 0.001. The dry cell weight was set to 0.28. All specific rates and yields were determined from the data over a period of 3–12 h

Changes in the medium composition over time are shown in Fig. 1C. The BY318-ATPase strain showed a significant decrease in the maximum concentration of glycerol of 48% compared with that of the BY318-Control strain (32.5 h). Additionally, the maximum concentration of acetate (32.5 h) was significantly lower, decreasing by 36%. In contrast, the maximum ethanol concentration (25 h) in the BY318-ATPase strain was significantly higher than that of the BY318-Control strain, increasing by 21%. These findings suggest that the biomass, glycerol, and acetate yields were reduced in the BY318-ATPase strain, whereas the ethanol yield was increased. The BY318-sceFBPase strain also showed significantly lower maximum glycerol (32.5 h) and acetate (32.5 h) concentrations (40% and 15% lower, respectively) than the BY318-Control strain. However, compared with that of the BY318-Control strain, the maximum ethanol concentration (25 h) did not significantly differ in the BY318-sceFBPase strain. The BY318-sceFBPase strain consumed glucose as fast as the BY318-Control strain did, but its OD_600_ and production of glycerol and acetate were considerably lower. Although the cause is unknown, the amount of intercellular metabolites and unmeasured by-products appeared to have increased. Furtheremore, the maximum ethanol concentration (25 h) was significantly higher in the BY318-opt_ecoFBPase strain (8%) than in the BY318-Control strain.

The specific consumption/production rate of each substance was determined from the slope of the plot of concentration change versus time-integrated dry cell weight (concentration during the logarithmic growth phase) (Table 1). First, the BY318-opt_ecoFBPase strain (10.5 ± 0.3 mmol/g-DCW/h) and BY318-ATPase strain (11.4 ± 1.0 mmol/g-DCW/h) exhibited higher specific glucose consumption rates than that of the BY318-Control strain (8.05 ± 0.42 mmol/g-DCW/h). Second, the BY318-opt_ecoFBPase strain (1.31 ± 0.02 mmol/g-DCW/h) and BY318-ATPase strain (1.19 ± 0.00 mmol/g-DCW/h) showed significantly increased specific glycerol production rates. Third, the BY318-opt_ecoFBPase strain (17.4 ± 0.6 mmol/g-DCW/h) and BY318-ATPase strain (20.3 ± 0.5 mmol/g-DCW/h) had significantly increased specific ethanol production rates. These results suggest that the BY318-opt_ecoFBPase and BY318-ATPase strains contained enhanced glycolytic systems with increased specific glucose consumption and ethanol production rates. The BY318-ATPase strain had a significantly lower glycerol yield, possibly because of the promotion of the downstream pathway of glycolysis to regenerate ATP, which suppressed glycerol production. Moreover, the BY318-opt_ecoFBPase and BY318-ATPase strains showed significantly reduced acetate yield, which may be attributed to the suppression of glycerol biosynthesis. Excess NADH generated by glycolysis may have led to suppression of the acetate biosynthesis pathway, including the reaction to regenerate NADH coupled with the oxidation of glyceraldehyde-3-phosphate to 1,3-biphosphoglycerate, resulting in the suppression of the acetate yield.

### Effect on growth and 2,3-BDO fermentation of laboratory strains introduced with a futile cycle

As the second target, we applied this strategy of ATP wasting to produce 2,3-BDO in the parent strain YHI030, which is deficient in pyruvate decarboxylase and heterologously overexpresses codon-optimized ALS from *L. plantarum*, ALDC from *L. lactis*, and BDH from *S. cerevisiae* (Additional file 1: Fig. S1). Strains were constructed by inducing the expression of each FBPase and ATPase under the *PGK1* promoter, as described in the Methods section.

**Fig. 2:**
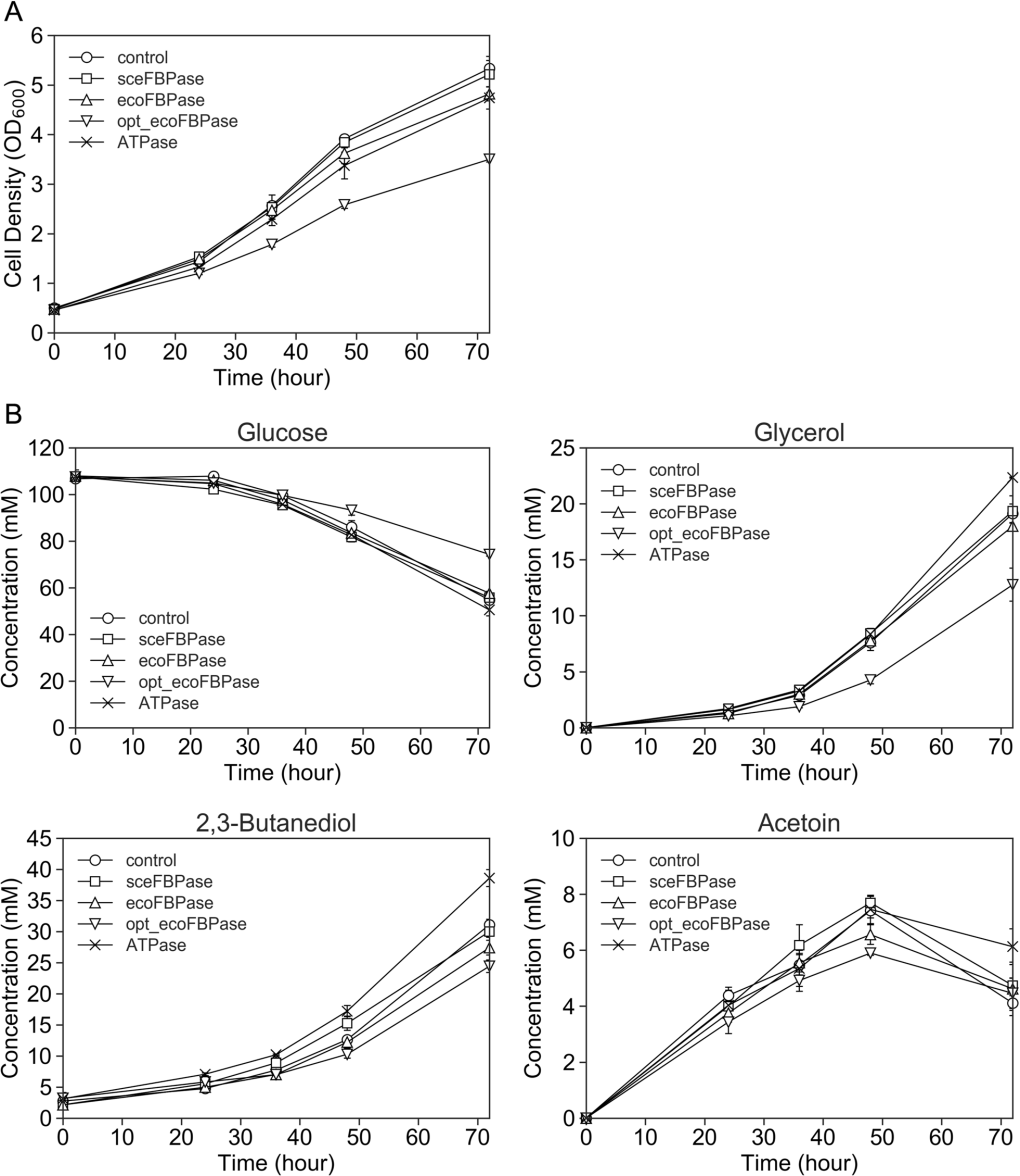
Fermentation profiles of YHI030 strains in flask-scale batch cultivation using synthetic medium. Time-course data of **A** cell density (OD_600_) and **B** concentrations (mM) of glucose, glycerol, ethanol, and acetate. YHI030 strains were cultured in 50 mL of medium in a 200 mL baffled flask shaken at 120 rpm. Data are expressed as the mean ± SD (n = 3)

Batch cultures of five strains, including the control strain, were conducted in SD medium, and a portion of the medium was collected after 0, 24, 36, 48, and 72 h. Cell growth and changes in the medium composition over time were examined by measuring the OD_600_ and extracellular metabolites (Fig. 2). The cells proliferated at 72 h, but exponential growth was considered limited to 48 h (Fig. 2A). The YHI030-opt_ecoFBPase strain showed a significantly lower specific growth rate of 0.036 ± 0.001 h^−1^ compared with that of the YHI030-Control strain at 0.044 ± 0.000 h^−1^, a decrease of 17% (Table 2). Figure 2B shows the changes in metabolites over time in the medium as quantified using HPLC and gas chromatography (GC). The specific consumption/production rates of each metabolite were calculated from the slope of the plot of concentration change versus time-integrated dry cell weight during the logarithmic growth phase (Table 2). At 72 h, the YHI030-opt_ecoFBPase strain exhibited the highest glucose concentration, which was 36% higher than that of the YHI030-Control strain. This strain also showed the lowest concentrations at 72 h for all metabolites measured except for acetoin, indicating that the YHI030-opt_ecoFBPase strain had the lowest fermentation capacity in terms of rate. Only the specific glucose consumption rate was significantly lower than that of the YHI030-Control strain, whereas the 2,3-BDO yield was 26% higher. These results indicate that the YHI030-opt_ecoFBPase strain had a slower growth rate and specific glucose consumption rate but a higher yield of 2,3-BDO at 72 h. The specific glucose consumption rate of the YHI030-opt_ecoFBPase strain was decreased, and that of the BY318-opt_ecoFBPase strain was increased (Table 1). The differences in the FBP and inorganic phosphate balance between the two strains with introduced FBPase may be related to variations in the native metabolic state of the parental strain and the presence or absence of the ethanol or 2,3-BDO biosynthetic pathways. The specific growth rate of the YHI030-sceFBPase strain was similar to that of the YHI030-Control strain, but the specific glucose consumption rate and specific 2,3-BDO production rate were significantly lower, and the 2,3-BDO yield was significantly higher by approximately 5% (Table 2).

**Table 2:** Growth rates and metabolite consumption and production rates and yields in YHI030 strains

	Specific growth rate (h^−1^)	Specific glucose consumption rate (mmol/g-DCW/h)	Specific 2,3-butanediol production rate (mmol (mmol/g-DCW/h)	Specific glycerol production rate (mmol (mmol/g-DCW/h)	Specific acetoin production rate (mmol (mmol/g-DCW/h)	2,3-Butanediol yield (mol (mol-glucose)^−1^)	Glycerol yield (mol (mol-glucose)^−1^)	Acetoin yield (mol (mol-glucose)^−1^)
Control	0.044 ± 0.000	0.920 ± 0.014	0.464 ± 0.017	0.316 ± 0.015	0.140 ± 0.020	0.505 ± 0.011	0.344 ± 0.022	0.152 ± 0.022
Scefbpase	0.044 ± 0.001	0.824 ± 0.019**	0.436 ± 0.015	0.319 ± 0.024	0.168 ± 0.018	0.529 ± 0.009*	0.388 ± 0.037	0.204 ± 0.025
Ecofbpase	0.043 ± 0.001	0.911 ± 0.017	0.436 ± 0.024	0.323 ± 0.007	0.133 ± 0.007	0.478 ± 0.025	0.355 ± 0.010	0.146 ± 0.006
Opt_ecoFBPase	0.036 ± 0.001***	0.797 ± 0.043**	0.506 ± 0.028	0.317 ± 0.041	0.164 ± 0.019	0.637 ± 0.059*	0.400 ± 0.068	0.207 ± 0.034
ATPase	0.042 ± 0.002	1.06 ± 0.06*	0.633 ± 0.029***	0.419 ± 0.002***	0.181 ± 0.023	0.598 ± 0.020**	0.397 ± 0.025*	0.170 ± 0.017

Asterisks indicate significant differences by two-tailed Student’s *t*-test, versus control. *p < 0.05, **p < 0.01, ***p < 0.001. The dry cell weight was set at 0.28

Specific rates and yields were determined from the data over a period of 0–48 h and 24–72 h, respectively

The YHI030-ATPase strain exhibited a comparable specific growth rate to the YHI030-Control strain but with a significant increase in the specific glucose consumption rate, specific 2,3-BDO and glycerol production rate, as well as 2,3-BDO and glycerol yield. Compared with the YHI030-Control strain, the YHI030-ATPase strain displayed a 15% increase in the specific glucose consumption rate, a 36% increase in the specific 2,3-BDO production rate, and an 18% increase in the 2,3-BDO yield. Additionally, the YHI030-ATPase strain exhibited superior fermentation performance compared with the BY318-ATPase strain, showing an increased specific glucose consumption rate and specific 2,3-BDO production rate while maintaining the specific growth rate (Table 2). Furthermore, the YHI030-ATPase strain displayed an elevated specific glycerol production rate and yield compared with the BY318-ATPase strain.

Because none of the YHI030 strains consumed glucose by 72 h, we collected a portion of the medium after 96, 120, 144, and 168 h from the YHI030-Control and the two improved strains, YHI030-opt_ecoFBPase and YHI030-ATPase. Prolonged culturing showed that the YHI030-ATPase strain consumed the most glucose and produced 2,3-BDO of 86.6 mM for 168 h (Additional file 1: Fig. S2). The 2,3-BDO yield of the YHI030-ATPase strain was also significantly higher than that of the YHI030-Control strain [0.833 ± 0.007 mol (mol-glucose) ^−1^ versus 0.813 ± 0.003 mol (mol-glucose) ^−1^], but that of the YHI030-opt_ecoFBPase strain was the same [0.799 ± 0.016 mol (mol-glucose) ^−1^]. These results indicate that the YHI030-ATPase strain improved both the productivity and yield of 2,3-BDO, even during the second half of the culture.

In ethanol biosynthesis, one glucose molecule yields 2 NADH through glycolysis, whereas 2 NADH are consumed in the ethanol biosynthetic pathway, thus maintaining the NADH/NAD^+^ balance (Additional file 1: Fig. S1). However, during 2,3-BDO biosynthesis via ALDC, only 1 NADH is consumed, leading to a 1 NADH surplus. Therefore, when 2,3-BDO yield is high (i.e., 2,3-BDO biosynthetic flux is activated), NADH/NAD^+^ is increased, and glycerol biosynthesis is stimulated to consume NADH. As a result, coexpression of an enzyme that converts NADH to NAD^+^, such as NADH oxidase, with ATPase may repress the glycerol yield. Previous studies reported that the expression of NADH oxidase from *L. lactis* in a pyruvate decarboxylase (PDC)-deficient BY4742 strain expressing ALS, ALDC, and BDH increased 2,3-BDO yield by 23.8% and reduced the glycerol yield by 65.3% [13]. These findings indicate that an ATPase-mediated reduction in the ATP concentration enhances glucose uptake and promotes the simultaneous production of ethanol and 2,3-BDO. FBPase influences the concentration of the sensitive metabolites FBP and inorganic phosphate, resulting in varied phenotypes among strains expressing different FBPases.

### Effects of introducing the futile cycle

**Fig. 3:**
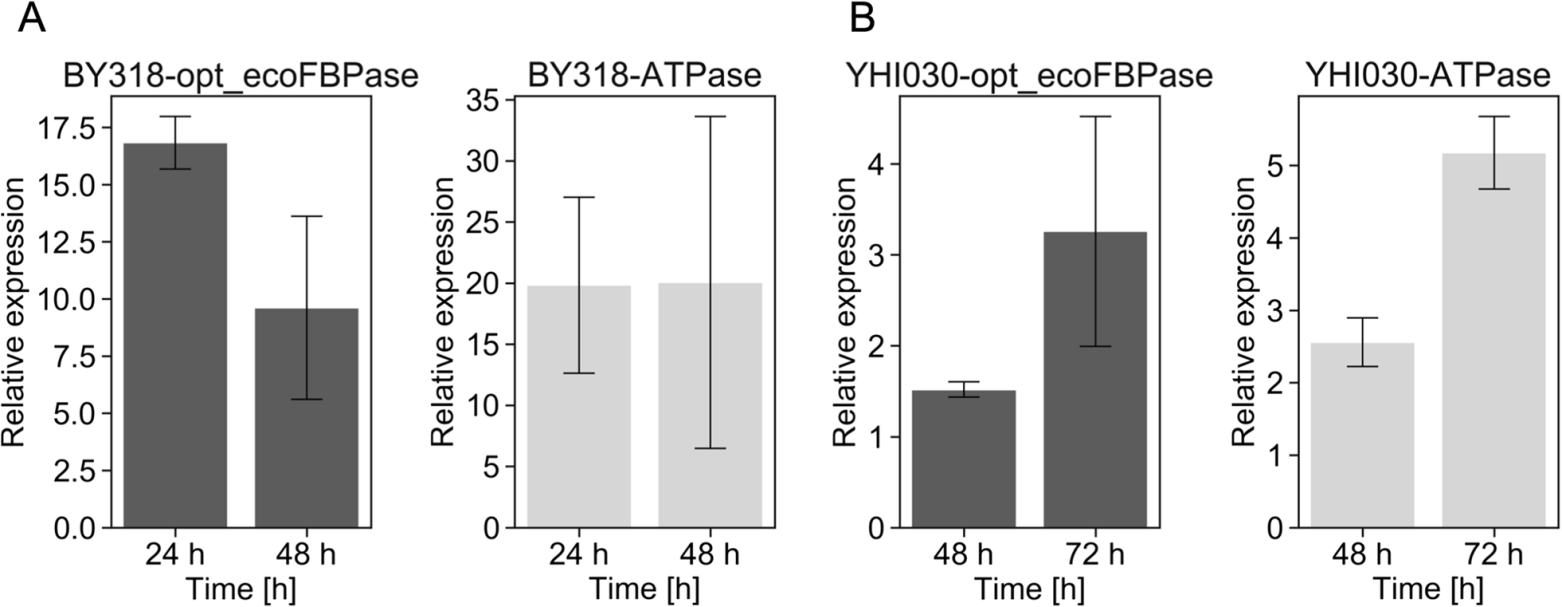
Expression levels of opt_ecoFBPase, ATPase in BY318 and YHI030 strains. Quantitative real-time PCR (qPCR) of relative expression levels of ecoFBPase and ATPase genes in (**A**) BY318 and (**B**) YHI030 strains. Expression levels were normalized to those of *ACT1*. Error bars represent the standard error of n = 3 independent experiments

In the laboratory strains BY318 and YHI030, the introduction of the futile cycle resulted in improved yields of ethanol and 2,3-BDO, respectively. A summary of the glucose consumption and ethanol and 2,3-BDO production yields is presented in Additional file 1: Fig. S3. Overexpression of opt_ecoFBPase in strain BY318 increased the specific glucose consumption rate by 30% and the specific ethanol production rate by 24%, whereas in strain YHI030, the specific glucose consumption rate decreased by 14% but the 2,3-BDO production rate increased by 9%. Similarly, overexpression of ATPase in the BY318 strain increased the specific glucose consumption rate by 42% and the specific ethanol production rate by 45%, while in YHI030 strain, the specific glucose consumption and specific 2,3-BDO production rates increased by 15% and 36%, respectively. These results suggest that the introduced futile cycle increased the yield of ethanol and 2,3-BDO. To further investigate the effect of opt_ecoFBPase and ATPase overexpression, we quantified gene expression levels at 24 and 48 h after incubation for BY318 and 48 and 72 h for YHI030. The cells were harvested, RNA extracted, and expression determined using quantitative polymerase chain reaction (qPCR). Target gene expression was normalized to the level of *ACT1* expression. In the BY318 strains, opt_ecoFBPase was 16.8- and 9.6-fold higher at 24 and 48 h, respectively, whereas ATPase expression was increased by approximately 20-fold (Fig. 3A). In the YHI030 strains, the expression of opt_ecoFBPase was 3.3-fold higher at 48 h and that of ATPase was 5.2-fold higher at 72 h (Fig. 3B). These results indicate that overexpression of these genes resulted in increased rates of specific glucose consumption and specific alcohol production. However, the gene expression levels did not necessarily correlated with ethanol or 2,3-BDO production, suggesting that the protein levels and activities of FBPase and ATPase may have been affected.

**Fig. 4:**
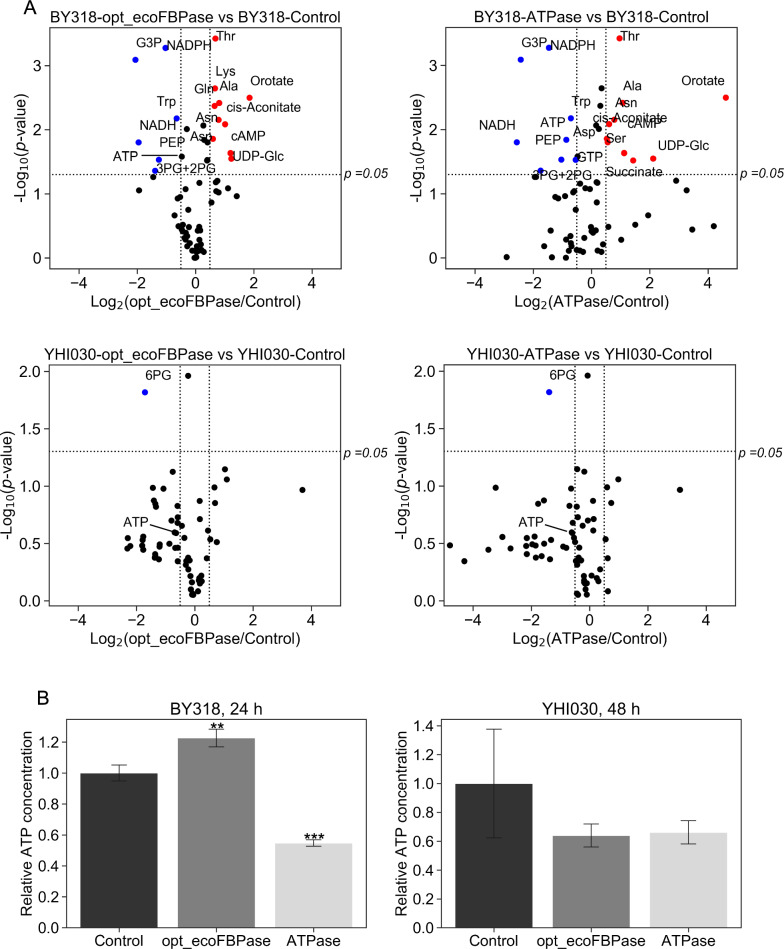
Comparison of intracellular metabolome and ATP levels in BY318 and YHI030 strains. **A** Comparison of metabolome profiles between the two strains using the volcano plot method. In these data, metabolites with an increase of at least 1.5-fold, a decrease of at least 0.66-fold, and a *t*-test *p*-value of 0.05 or less were considered significantly increased (shown in red) or decreased (shown in blue). **B** Relative ATP concentration of BY318 and YHI030 strains expressing either opt_ecoFBPase or ATPase compared to control strains. Error bars indicate the standard deviation, and asterisks indicate the results of the two-sided *t*-test (*p < 0.05, **p < 0.01, ***p < 0.001, n = 3)

Finally, metabolomic analysis was conducted to investigate changes in intracellular metabolites, including ATP, in the strains. Cells were harvested at 24 h for BY318-opt_ecoFBPase and BY318-ATPase, and at 48 h for YHI030-opt_ecoFBPase and YHI030-ATPase. Volcano plot analysis was performed for 66 metabolites (Fig. 4A) to visualize differences between a control strain and strains expressing FBPase or ATPase. The BY318-opt_ecoFBPase and BY318-ATPase strains showed significantly lower levels of NADH, possibly because of the increased rate of ethanol production and the use of NADH to produce more ethanol. In contrast, some metabolites (such as orotate and UDP-Glc) accumulated at high levels in both strains. ATP levels were significantly reduced by 45% in the BY318-ATPase strain (Fig. 4B, Additional file 2: Table S1), whereas no reduction was observed in the BY318-opt_ecoFBPase strain. Because the total amount of intracellular metabolites in the BY318-opt_ecoFBPase strain was approximately 1.5-fold higher than that in the BY318-Control strain (Additional file 2: Table S1), the ATP levels may not have been reduced. Metabolite changes in the YHI030-opt_ecoFBPase and YHI030-ATPase strains did not greatly differ from those in the YHI030-Control strain (Fig. 4A), but NADH levels were lower (Additional file 2: Table S2), possibly because only one NADH is used to produce 2,3-BDO. In addition, metabolites such as 3-phosphoglycerate, 2-phosphoglycerate, and phospho*enol* pyruvate, which are produced in the second half of glycolysis, were reduced (Fig. 4A, Additional file 2: Table S2). ATP concentrations were significantly reduced by 36% and 34% in the YHI030-opt_ecoFBPase and YHI030-ATPase strains, respectively (Fig. 4B). Intracellular ATP concentrations in these strains were similar to the adenylate energy charge (Additional file 1: Fig. S4). These results suggest that ATPase can reduce the ATP concentration to enhance glucose uptake and alcohol production in the form of ethanol and 2,3-BDO. The phenotypes differed among strains expressing different FBPases because FBPase affected the concentration-sensitive metabolites FBP and inorganic phosphate, indicating that FBPase activity can be manipulated to further enhance the alcohol production capacity of yeast.

## Discussion

Although ATP can stimulate cellular growth, excessive ATP levels can hamper the productivity of metabolic pathways [25]. In such cases, ATP consumption can be artificially boosted to trigger favorable metabolic responses while limiting proliferation [32]. Artificially increasing ATP consumption is a promising metabolic engineering strategy for enhancing microbial metabolite production [20, 26, 27, 33–39]. For instance, engineered ATP consumption was incorporated into *n*-butanediol production in cyanobacteria to boost metabolic flux to target pathways [35]. More recently, introducing *E. coli* F1-ATPase expression in *S. cerevisiae* led to improved ethanol production [18]. The ATP-wasting cycle can be induced by constitutively expressing ATPase or by creating a more complex futile cycle that causes net ATP hydrolysis. Thus, we overexpressed several FBPases and ATPases as futile cycles in *S. cerevisiae* strains and examined the effects on bioalcohol production based on the growth rate, glucose consumption rate, and alcohol production rate when the cells were grown on minimal synthetic media.

To introduce the futile cycle, we used ecoFBPase from *E. coli*, opt_ecoFBPase codon-optimized from yeast, sceFBPase from *S. cerevisiae*, and ATPase (*E. coli* subunits α, β, and γ linked via a 2A sequence to enable polycistronic expression). Overexpression of these genes under a strong *PGK1* promoter resulted in increased ethanol production rates in laboratory strains expressing either opt_ecoFBPase or ATPase (Fig. 1C, Table 1). Expression of ATPase also typically impacts substrate uptake rates. For instance, in *E. coli*, expression of the F1 ATPase gene elevated glucose uptake rates, contributing to increased specific productivity [33]. Forcing ATP consumption can significantly increase glucose uptake rates and target compound production rates [18–20, 26, 34, 40]. The rate of glucose consumption was increased, suggesting that opt_ecoFBPase and ATPase overexpression increased the specific production rate via enhanced substrate uptake. Furthermore, the specific glycerol yield decreased in both the BY318-opt_ecoFBPase and BY318-ATPase strains (Fig. 1C, Table 1), possibly because of an accelerated metabolic pathway downstream in glycolysis for ATP regeneration. Although ethanol yield was not improved in the BY318 strain, it may be improved by performing nitrogen-limited culture to suppress biomass synthesis under anaerobic conditions, similar to the results observed in a previous study [18].

Our results support that the metabolic engineering strategy of introducing opt_ecoFBPase and ATPase can increase the ethanol production rate in a laboratory strain. This method can also be extended to the production of other bioalcohols. The YHI030-opt_ecoFBPase strain did not significantly enhance the specific production rate of 2,3-BDO, but the yield of 2,3-BDO was increased by 26% at 72 h (Fig. 2B, Table 2). In contrast, in the YHI030-ATPase strain, the specific 2,3-BDO production rate and yield were increased by 36% and 18%, respectively (Fig. 2B, Table 2). YHI030 was cultured in an SD medium and showed a yield of 0.41 g g^−1^ glucose in a previous study [15]. To our knowledge, the highest reported yield of 2,3-BDO is 0.54 g g^−1^ glucose in YPH499/dPdAdG/BD6-10/FLO1in a fed-batch cultivation using yeast-peptone-dextrose (YPD) medium [41]. However, previous studies showed that YPD medium provides higher yields than SC medium (SD medium containing amino acids) [13, 14]. Among the reported cases, YHI030 showed the highest yield in fed-batch culture in SD medium. We calculated the yields of 2,3-BDO (g g^−1^ glucose) from the specific consumption rates of glucose, with YHI030-Control, YHI030-opt_ecoFBPase, and YHI030-ATPase showing values of 0.25, 0.32, and 0.30 g g^−1^ glucose, respectively. Based on these results, the 2,3-BDO yields in YHI030-opt_ecoFBPase and YHI030-ATPase strains can exceed 0.54 g g^−1^ glucose when the cells are grown in fed-batch culture with YPD medium.

The expression of ATPase in the strains increased ATP consumption and reduced the growth rate and biomass (Figs. 1, 2A, Tables 1, 2). Therefore, there is a trade-off between biomass and target compound production, and either yield or production must be prioritized depending on the application. Separating proliferation from material production has been experimentally and theoretically validated and is effective for target compound production [18, 42]. In general, FBPase or ATPase overexpression may not be suitable for a scaled-up production. However, YHI030-ATPase enhanced both the yield and production of 2,3-BDO, perhaps because the expression of 2,3-BDO biosynthetic pathway genes in this strain is regulated by two strong promoters, *PGK1* and *ADH1*. These promoters strongly pull pyruvate toward 2,3-BDO production rather than towards the tricarboxylic acid cycle. Therefore, increasing the rate of bioalcohol production by expressing ATPase may improve even the yield in substance-producing strains, such as YHI030, constructed under a strong expression promoter. It may be possible to fine-tune the expression or timing of ATPases to enhance the production of target compounds, while maintaining cell growth.

Our results demonstrate the feasibility of enhancing alcohol yields, including the yields of ethanol and 2,3-BDO, by imposing ATP consumption on *S. cerevisiae*. Applying this ATP-wasting strategy to industrial strains is an issue that needs to be addressed in the future. Further research on the impact of ATP depletion in *S. cerevisiae* with alternative substrates (e.g., sucrose) and optimizing the production of other beneficial metabolites linked to ATP energy generation is needed. As different laboratory strains and culture conditions may influence yield and product formation, metabolome analysis of metabolite accumulation would be valuable for metabolic engineering of *S. cerevisiae* cells to discern the potential for enforced ATP consumption.

## Conclusions

To enhance the capacity of *S. cerevisiae* to produce alcohol by introducing a futile cycle, we initially selected three FBPases (ecoFBPase, sceFBPase, and opt_ecoFBPase) and an ATPase as futile cycles for overexpression in the laboratory strain BY318. The specific ethanol consumption rates of the BY318-opt_ecoFBPase and BY318-ATPase strains were notably increased. Moreover, when this strategy was implemented in a 2,3-BDO-producing strain (YHI030), the yield of 2,3-BDO per glucose consumption increased in the YHI030-opt_ecoFBPase and YHI030-ATPase strains, along with an increase in the specific 2,3-BDO production rate. The intracellular ATP concentration was significantly reduced in these strains. Thus, the ATP consumption strategy enhanced the performance of multiple strains.

## Methods

### Strains, media, and culture conditions

The strains used in this study were derived from two parent strains, the *S. cerevisiae* laboratory strain BY318 and the 2,3-BDO-producing strain YHI030 [15], as indicated in Table 3. SD medium composed of 6.7 g/L yeast nitrogen base without amino acids and 20 g/L glucose was used for cell cultures. The agar medium contained 20 g/L agar. The medium was supplemented with 20 mg/L histidine, 40 mg/L tryptophan, 30 mg/L lysine hydrochloride, and 20 mg/L uracil when necessary. Lysogeny broth medium containing 10 g/L bacto-tryptone, 5 g/L bacto-yeast extract, and 10 g/L NaCl was used to culture *E. coli* cultures. If needed, 50 mg/L ampicillin was added.

**Table 3:** Strains used in this study

Strain name	Genotype	Source
BY318	*MATa ura3-52*	National BioResource Project
BY318-Control	BY318 (pGK426)	This study
BY318-sceFBPase	BY318 (pGK426-sceFBPase)	This study
BY318-ecoFBPase	BY318 (pGK426-ecoFBPase)	This study
BY318-opt_ecoFBPase	BY318 (pGK426-opt_ecoFBPase)	This study
BY318-ATPase	BY318 (pGK426-atpAGD)	This study
YPH499	*MATa ura3-52 lys2-801 ade2-101 trp1-Δ63 his3-Δ200 leu2-Δ1*	Stratagene / Agilent Technologies
YSM021 (PDCΔ)	YPH499 *pdc1Δ pdc5Δ pdc6Δ MTH1-ΔT*(*L165F*)	Ishii et al. (2018)
YSM046 (PDCΔ + evolved)	A laboratory-evolved yeast strain derived from PDCΔ (YSM021) strain	Ishii et al. (2018)
YHI030	YSM046 (pATP422-alsLpOp-aldcLlOp/pAT425-BDH1)	Ishii et al. (2018)
YHI030-Control	YHI030 (pGK426)	This study
YHI030-sceFBPase	YHI030 (pGK426-sceFBPase)	This study
YHI030-ecoFBPase	YHI030 (pGK426-ecoFBPase)	This study
YHI030-opt_ecoFBPase	YHI030 (pGK426-opt_ecoFBPase)	This study
YHI030-ATPase	YHI030 (pGK426-atpAGD)	This study

### Plasmid construction

Three FBPases and one ATPase were integrated into the BY318 and YHI030 strains, respectively, to generate overexpressing strains. Plasmids were constructed using the pGK426 expression vector [43] as a template (Additional file 2: Table S3). PCR was performed using a KOD FX Neo (TOYOBO, Osaka, Japan), and T4 DNA Ligase (New England Labs, Ipswich, MA, USA) and T4 Polynucleotide Kinase (New England Labs) were used for ligation. The DNA was purified with the Wizard SV Gel and PCR Clean-Up System (Promega, Madison, WI, USA). Sequences were analyzed by Eurofins service.

### Construction of pGEM-T-easy-sceFBPase and pGEM-T-easy-ecoFBPase

Genomic DNA from *S. cerevisiae* S288C and *E. coli* DH5α (TaKaRa, Shiga, Japan) was used as a template to amplify target gene fragments with double-ended restriction enzyme sequences (NheI and BglII) via PCR using the primer sets NheI_fbpSc_fw/BglII_fbpSc_rv and NheI_fbpEc_fw/BglII_fbpEc_rv (Additional file 2: Table S4). Specifically, sceFBPase (*FBP1* from *S. cerevisiae*) and ecoFBPase (*fbp* from *E. coli*) were amplified from the genomes of their respective organisms. To add smooth ends to the resulting gene fragments, deoxyriboadenine was added, followed by ligation to the pGEM-T-easy vector and clone selection. Plasmids were extracted from each clone, followed by sequence confirmation to construct pGEM-T-easy-sceFBPase and pGEM-T-easy-ecoFBPase derived from the pGK426 plasmids.

### Construction of pGK426-sceFBPase and pGK426-ecoFBPase

The plasmids pGEM-T-easy-sceFBPase and pGEM-T-easy-ecoFBPase, along with the plasmid pGK426, were subjected to restriction enzyme digestion using NheI and BglII. The resulting FBPase gene fragments were ligated into the pGK426 vector by incubating the reaction mixture at 37 °C for 30 min. The desired plasmid was obtained by transforming *E. coli* with the ligation mixture, followed by plasmid extraction.

### Construction of pGK426-opt_ecoFBPase

The plasmid pGK426-opt_ecoFBPase was engineered to enable the expression of the opt_ecoFBPase gene that has been optimized for *S. cerevisiae* by codon optimization. The codon optimization algorithm was developed by Raab et al. [44], and gene synthesis was carried out by GeneArt (Thermo Fisher Scientific, Waltham, MA, USA). The target plasmid, pGK426-opt_ecoFBPase, was constructed by digesting the synthetic gene fragment and pGK426 using restriction enzymes NheI and BglII, followed by ligation and transformation into *E. coli*.

### Construction of pGK426-atpAGD

A plasmid named pGK426-atpAGD was engineered using the 2A sequence, which enables the expression of ATPase from a single mRNA. The plasmid was designed based on the method described by Zahoor et al. [18]. Initially, primers (pGK426_BglII_fw and pGK426_NheI_rv) were designed in opposite directions from the *PGK1* promoter and *PGK1* terminator, respectively, using pGK426 as a template. The DNA fragments for generating the vectors were obtained using inverse PCR. Primers containing 2A sequences between each *atpA*, *atpG*, and *atpD* were designed, and three fragments were obtained using the primer sets pGK426_NheI_atpA_fw/ERBV-1_atpA_rv, ERBV-1_atpG_fw/P2A_atpG_rv, and P2A_atpD_fw/pGK426_BglII_atpD_rv (Additional file 2: Table S4). The four fragments were assembled using Gibson Assembly Master Mix (New England Labs) to transform *E. coli*. Plasmids were recovered from the transformants of *E. coli* and sequenced with atpA_sq1, atpA_sq2, atpG_sq3, and atpD_sq4 to confirm successful production.

### Cultivation conditions

#### BY318-based strains

Five strains of BY318 (BY318-Control, BY318-sceFBPase, BY318-ecoFBPase, BY318-opt_ecoFBPase, and BY318_ATPase) were propagated in the pre-culture stage in baffled 200 mL Erlenmeyer flasks containing 5 mL SD medium while shaking (30 °C, 150 rpm, n = 3) for approximately 24 h. The pre-cultured cells were transferred into baffled 200 mL Erlenmeyer flasks containing 50 mL SD medium with an initial OD_600_ of 0.05 and incubated for 16 h (30 °C, 120 rpm, n = 3). This pre-culture solution was transferred into 200 mL baffled Erlenmeyer flasks containing 50 mL SD medium with an initial OD_600_ of 0.05 and incubated while shaking (30 °C, 120 rpm, n = 3), except for BY318-sceFBPase and BY318-ecoFBPase, which were cultured at n = 8.

#### YHI030-based strains

Five YHI030-derived strains (YHI030-Control, YHI030-sceFBPase, YHI030-ecoFBPase, YHI030-opt_ecoFBPase, and YHI030_ATPase) were inoculated into Malton tubes containing 5 mL of SD medium at an OD_600_ of 3 and cultured for 2 days (30 °C, 150 rpm, n = 3). The cells were transferred into fresh Malton tubes containing 5 mL of SD medium with an initial OD_600_ of 0.5 and incubated for approximately 2 days (30 °C, 150 rpm, n = 3). The resulting pre-culture solution was inoculated into fresh Malton tubes containing 5 mL of SD medium with an initial OD_600_ of 0.5 and incubated while shaking (30 °C, 150 rpm, n = 3).

#### RNA extraction and qPCR

Following 24 and 48 h of incubation of the BY318 strains (BY318-Control, BY318-opt_ecoFBPase, and BY318-ATPase) and 48 h of incubation of the YHI030 strains (YHI030-Control, YHI030-opt_ecoFBPase, and YHI030-ATPase), the cells were harvested at OD_600_ = 3.0 after 48 and 72 h of incubation, respectively. The cells were centrifuged at 17,970 × *g* for 3 min and washed with TE buffer. After discarding the supernatant, 200 μL RNA buffer (0.8 M sorbitol, 100 mM EDTA, 14 mM β-mercaptoethanol) and zymolyase-20 T (1000 U/mL) (Nacalai Tesque, Kyoto, Japan) were added to the culture, which was incubated at 30 °C for 30 min. The purified RNA was subjected to cDNA synthesis using the PrimeScript™ RT Reagent Kit (Perfect Real Time, TaKaRa). The target genes were quantified using TB Green™ Premix Ex Taq^™^ (Tli RNaseH Plus, TaKaRa) and a StepOnePlus real-time PCR system. Amplification was performed using the following primer sets: ACT1_for_RT-PCR_F/ACT1_for_RT-PCR_R for ACT1 (control gene), ATPase_for_RT-PCR_F/ATPase_for_RT-PCR_R for ATPase, and optFBPase_for_RT-PCR_F/optFBPase_for_RT-PCR_R for FBPase. Target genes were quantified based on the obtained amplification signals.

#### Measurement of cell density, extracellular/intracellular metabolites, and energy charge

Cell density was determined using UV–visible spectrophotometry (UV-1700, Shimadzu, Kyoto, Japan) by calculating the OD_600_. The concentrations of carbon sources (glucose, glycerol, and acetate) in the medium were measured using HPLC (HPLC Prominence, Shimadzu) with a refractive index detector [45].

The concentrations of ethanol, 2,3-BDO, diacetyl, and acetoin were analyzed via GC equipped with a frame ionization detector (GC-2025, Shimadzu) with slight modifications from the previous study [46]. After centrifugation (5 min, 17,970 ×*g*, 4 °C), the supernatant was filter-sterilized through a 0.45 µm pore size Cosmo filter W. The column used was a DB-WAX (0.32 mm, 60 m, 0.25 µm, Agilent Technologies, Santa Clara, CA, USA). 3-Methyl-1-butanol was used as an internal standard. To prepare the samples, 50 µL of 0.1% 3-methyl-1-butanol was mixed with an appropriately diluted sample. An external calibration curve was generated, and concentrations were quantified.

Cells were collected by rapid filtration and quenched using methanol containing 5 µM d-camphor sulfonic acid as previously described [47]. Extraction of intracellular metabolites was performed using the methanol-chloroform-water method [47], whereby 640 µL Milli-Q water and 1.6 mL chloroform were added to the collected sample, and the samples were vortexed and sonicated for 1 min each. The mixture was centrifuged (4 °C, 1450 × *g*, 20 min) in an oscillating rotor (Eppendorf Himac Technologies, Ibaraki, Japan). After centrifugation, the tubes were incubated at 4 °C for 10 min, after which 250 µL of the supernatant was dispensed into five 1.5 mL tubes and dried at 25 °C under reduced pressure using a centrifugal evaporator CVE-3110 (EYELA, Tokyo, Japan). The dried sample was dissolved in 50 µL of Milli-Q water, centrifuged at 20,630 ×*g* for 5 min at 25 °C, and the supernatant was analyzed for intracellular metabolites using LCMS-8060X (Shimadzu) in negative mode for ion-pair liquid chromatography-tandem mass spectrometry using the LC/MS/MS Method Package for Primary Metabolites Ver. 2 (Shimadzu). A MASTRO2 C18 (GLC) column (150 mm, 2.1 mm, 3 µm, Shimadzu) was used, with 10 mM tributylamine and 15 mM acetate as eluent A and methanol as eluent B. The column temperature was set to 40 °C, and the flow rate was 1.5 mM/min. The injection volume was 1 µL, and the gradient was initiated at 0% B, increased to 25% B for 8 min, and then increased to 98% B for 12 min. The concentration was maintained at 98% B for 3 min before returning to 0% B. Negative ion mode was used for mass spectrometry analysis, with the electrospray voltage set to 4.0 kV, desolvation line set to 250 °C, heating block temperature set to 400 °C, nebulizing gas (N_2_) flow rate of 3.0 L/min, drying gas (N_2_) flow rate of 15.0 L/min, and collison-induced dissociation gas (Ar) pressure of 0.27 Mpa. d-Camphor sulfonic acid was used as an internal standard.

The adenylate energy charge (EC) is an index of the energy status of biological cells. The EC was calculated using the following equation:

$${\text{EC = [ATP] + 0}}{.5} \times {{[{\text{ADP]}}} \mathord{\left/ {\vphantom {{[{\text{ADP]}}} {[{\text{ATP}}}}} \right. \kern-0pt} {[{\text{ATP}}}}] + [{\text{ADP] + [AMP]}}$$

### Supplementary Information

**Additional file 1: Fig. S1.** 2,3-butanediol (2,3-BDO) biosynthetic pathways in YHI030. A pyruvate decarboxylase (PDC)-deficient (*PDCΔ*) strain (containing the MTH1-ΔT allele and subjected to laboratory evolution) was used to ensure the pulling of pyruvate carbon flux and higher 2,3-BDO production. Acetolactate decarboxylase (ALDC) and butanediol dehydrogenase (BDH) were additionally expressed to avoid clogging the carbon flux towards 2,3-BDO biosynthesis [15]. **Fig. S2.** Prolonged fermentation profiles of some YHI030 strains in flask-scale batch cultivation using synthetic medium. Time-course data for **A** cell density (OD_600_) and **B** concentrations (mM) of glucose, glycerol, ethanol, and acetate. YHI030 strains were cultured in 50 mL of medium in a 200 mL baffled flask shaken at 120 rpm. Data are expressed as the mean ± SD (n = 3). **Fig. S3.** Effects of expression levels of opt_ecoFBPase, ATPase in BY318 and YHI030 strains. Specific rate of glucose consumption and ethanol production in BY318 strains and that in YHI030 strains during flask-scale batch cultivation using a synthetic medium. Each rate was determined from the exponential growth data. Error bars indicate standard deviation, and asterisks indicate the results of the two-sided *t*-test (*p < 0.05, **p < 0.01, ***p < 0.001, n = 3). **Fig. S4.** Comparison of adenylate energy charge of BY318 and YHI030 strains. Adenylate energy charge (EC) of BY318 and YHI030 strains expressing either opt_ecoFBPase or ATPase, in addition to the control strain. Error bars indicate the standard deviation, and asterisks indicate the results of a two-sided *t*-test (*p < 0.05, **p < 0.01, ***p < 0.001, n = 3).

**Additional file 2: Table S1.** Metabolite concentration in BY318 strains normalized to d-camphor sulfonic acid (set = 1). **Table S2.** Metabolite concentration in YHI030 strains normalized to d-camphor sulfonic acid (set = 1). **Table S3.** Plasmids used in this study. **Table S4.** Primers used in this study.

## Data Availability

All data for this study are included in this manuscript.

## Abbreviations

ALDC: Acetolactate decarboxylase
ALS: Acetolactate synthase
BDH: Butanediol dehydrogenase
BDO: Butanediol
EC: Energy charge
ERBV-1: Equine rhinitis B virus
F6P: Fructose 6-phosphate
FBP: Fructose 1,6-bisphosphate
GC: Gas chromatography
HPLC: High-performance liquid chromatography
OD: Optical density
PDC: Pyruvate decarboxylase
PTV: Porcine teschovirus
qPCR: Quantitative real-time PCR
SD: Synthetic dextrose
YPD: Yeast-peptone-dextrose
