# Supplementary figures and images for: Improvement of ethanol and 2,3-butanediol production in Saccharomyces cerevisiae by ATP wasting
Source: Microb Cell Fact. 2023 Oct 8;22:204. doi: 10.1186/s12934-023-02221-z (PMC10560415; doi:10.1186/s12934-023-02221-z)

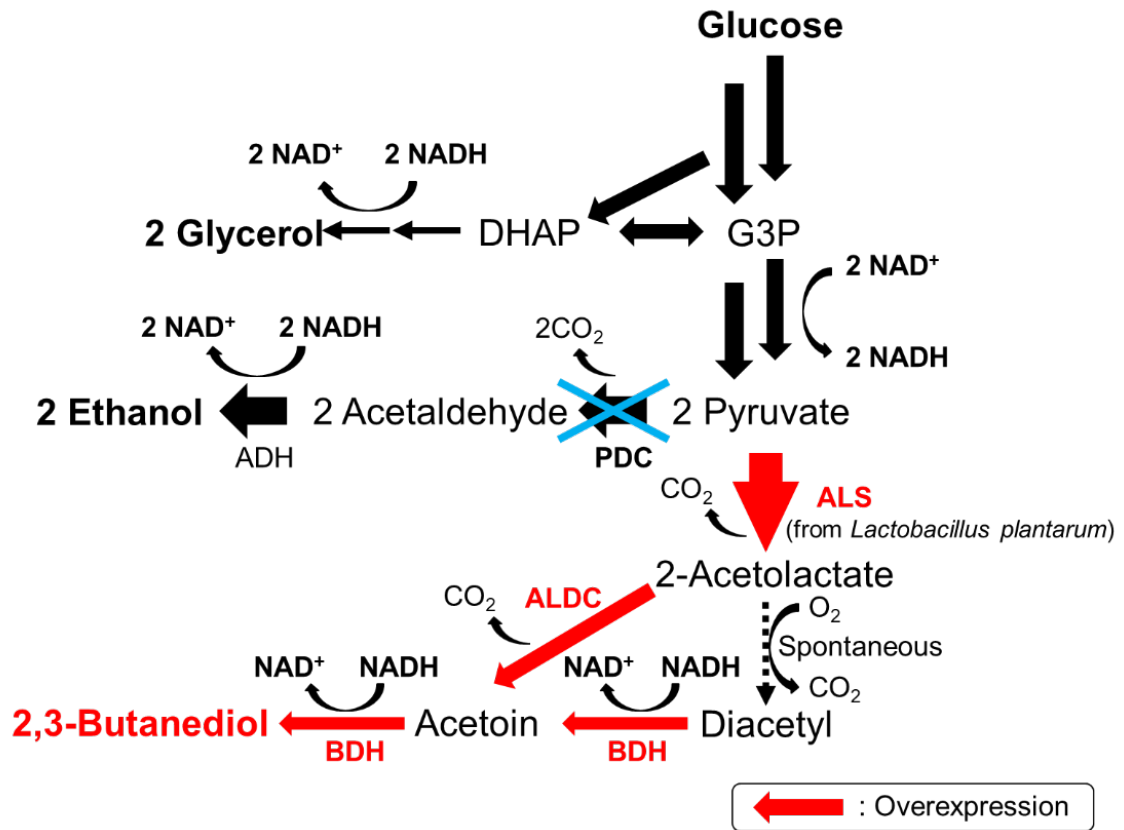

A

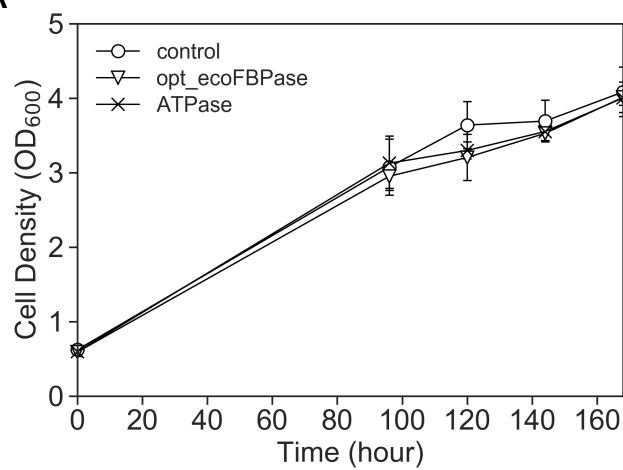

B

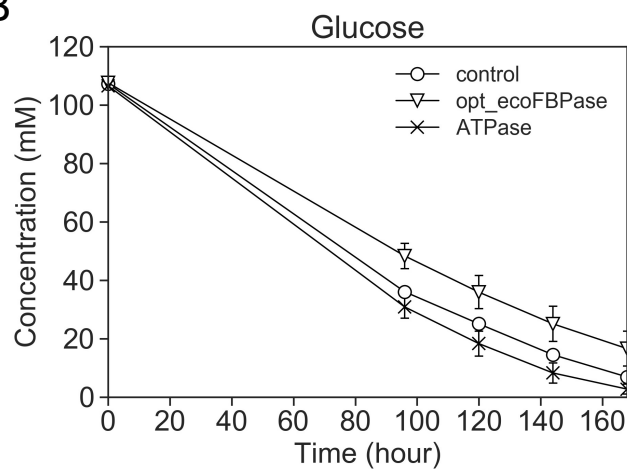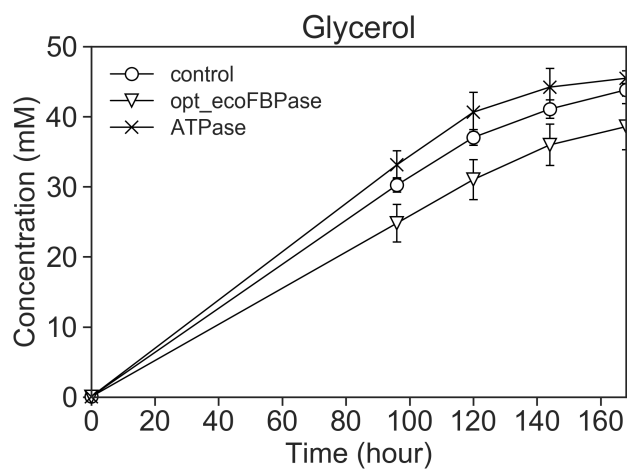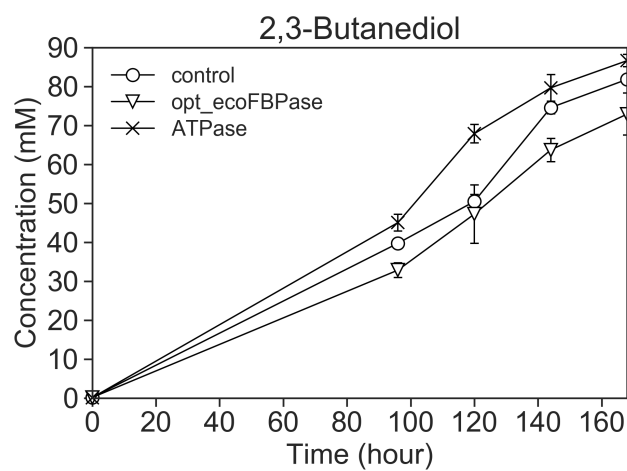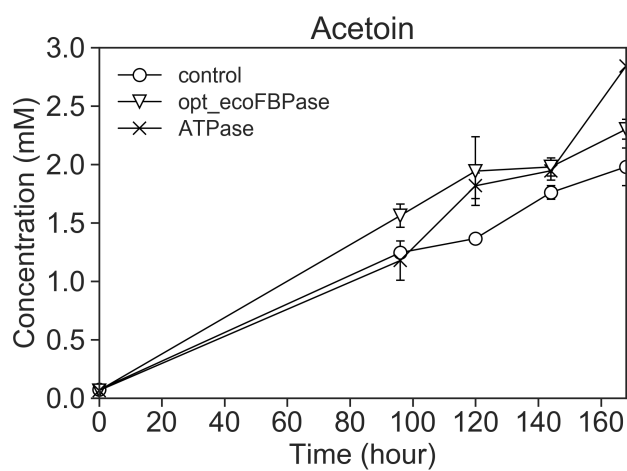

Fig. S3

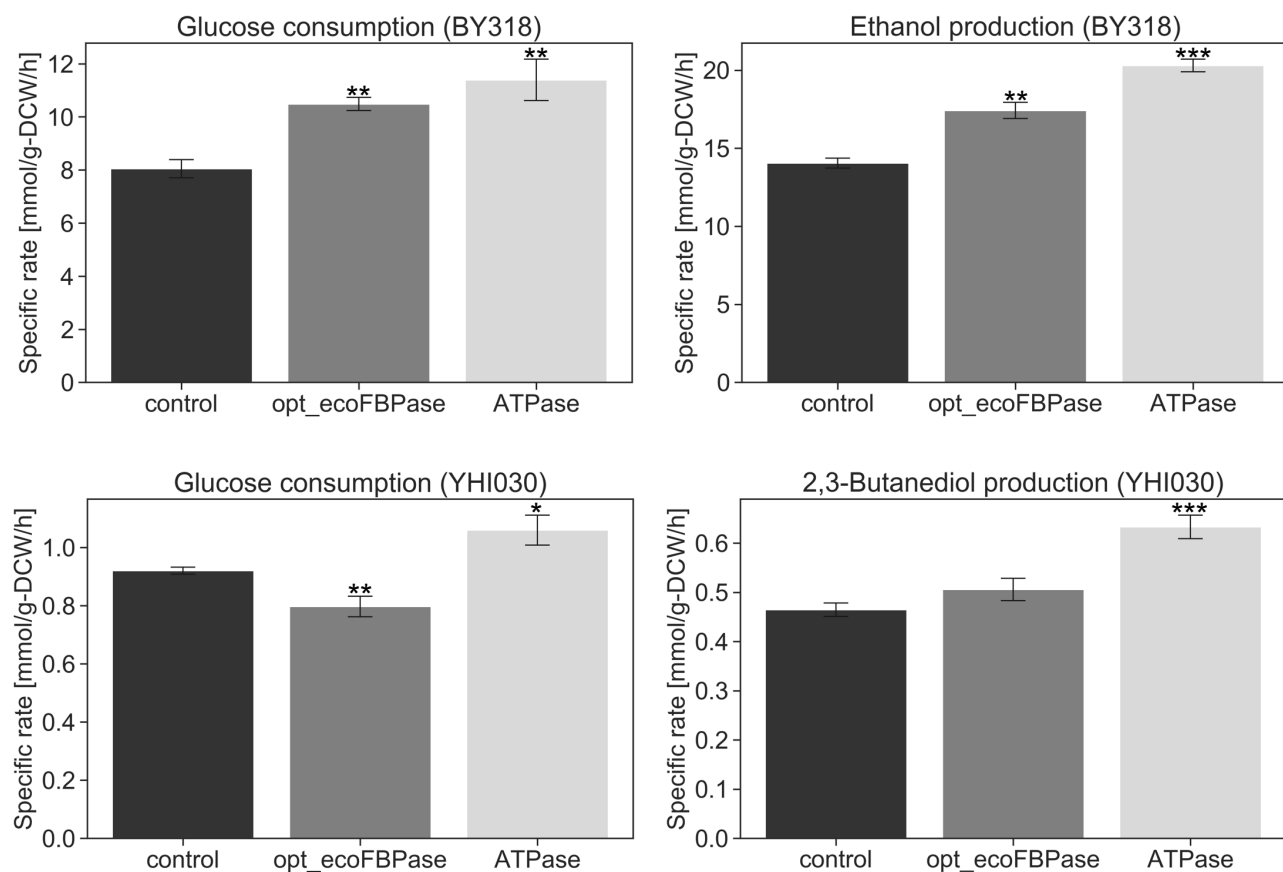

A

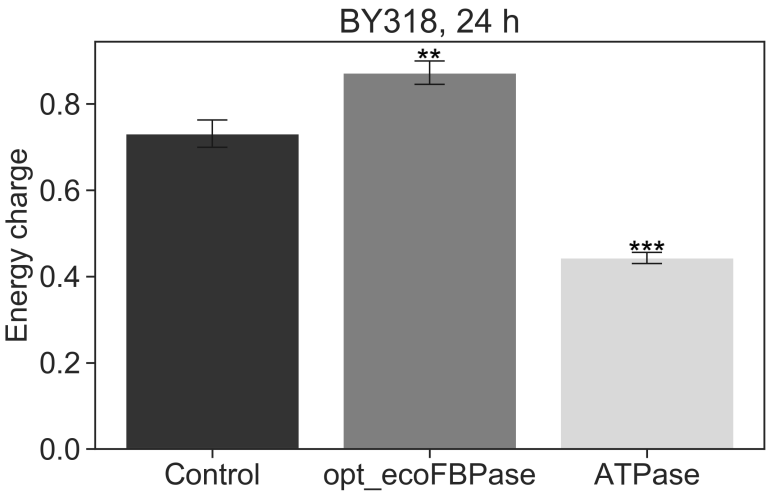

B

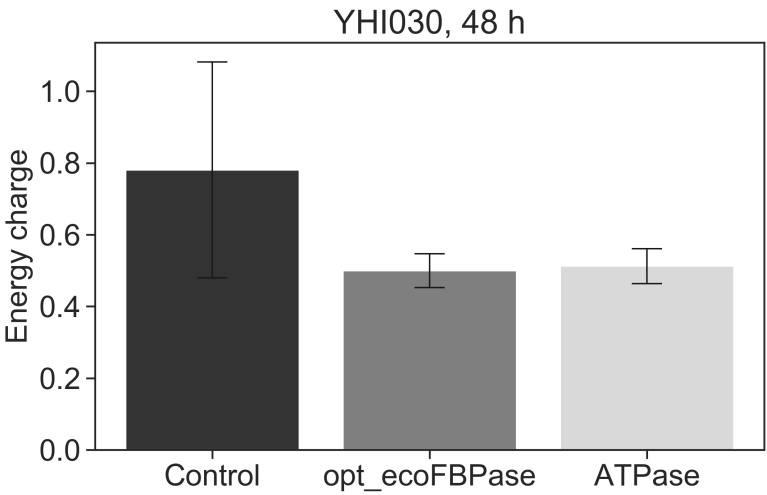

Supplement: Supplementary file 1 — Additional file 1: Fig. S1. 2,3-butanediol (2,3-BDO) biosynthetic pathways in YHI030. A pyruvate decarboxylase (PDC)-deficient (PDCΔ) strain (containing the MTH1-ΔT allele and subjected to laboratory evolution) was used to ensure the pulling of pyruvate carbon flux and higher 2,3-BDO production. Acetolactate decarboxylase (ALDC) and butanediol dehydrogenase (BDH) were additionally expressed to avoid clogging the carbon flux towards 2,3-BDO biosynthesis [15]. Fig. S2. Prolonged fermentation profiles of some YHI030 strains in flask-scale batch cultivation using synthetic medium. Time-course data for A cell density (OD600) and B concentrations (mM) of glucose, glycerol, ethanol, and acetate. YHI030 strains were cultured in 50 mL of medium in a 200 mL baffled flask shaken at 120 rpm. Data are expressed as the mean ± SD (n = 3). Fig. S3. Effects of expression levels of opt_ecoFBPase, ATPase in BY318 and YHI030 strains. Specific rate of glucose consumption and ethanol production in BY318 strains and that in YHI030 strains during flask-scale batch cultivation using a synthetic medium. Each rate was determined from the exponential growth data. Error bars indicate standard deviation, and asterisks indicate the results of the two-sided t-test (*p < 0.05, **p < 0.01, ***p < 0.001, n = 3). Fig. S4. Comparison of adenylate energy charge of BY318 and YHI030 strains. Adenylate energy charge (EC) of BY318 and YHI030 strains expressing either opt_ecoFBPase or ATPase, in addition to the control strain. Error bars indicate the standard deviation, and asterisks indicate the results of a two-sided t-test (*p < 0.05, **p < 0.01, ***p < 0.001, n = 3). [file 12934_2023_2221_MOESM1_ESM.pdf]
