# Supplementary material for: Improvement of ethanol and 2,3-butanediol production in Saccharomyces cerevisiae by ATP wasting
Source: Microb Cell Fact. 2023 Oct 8;22:204. doi: 10.1186/s12934-023-02221-z (PMC10560415; doi:10.1186/s12934-023-02221-z)
Supplement: Supplementary file 2 — Additional file 2: Table S1. Metabolite concentration in BY318 strains normalized to d-camphor sulfonic acid (set = 1). Table S2. Metabolite concentration in YHI030 strains normalized to d-camphor sulfonic acid (set = 1). Table S3. Plasmids used in this study. Table S4. Primers used in this study. [file 12934_2023_2221_MOESM2_ESM.docx]

**Additional file information**

**Fig. S1 2,3-butanediol (2,3-BDO) biosynthetic pathways in YHI030.**

A pyruvate decarboxylase (PDC)-deficient (*PDCΔ*) strain (containing the MTH1-ΔT allele and subjected to laboratory evolution) was used to ensure the pulling of pyruvate carbon flux and higher 2,3-BDO production. Acetolactate decarboxylase (ALDC) and butanediol dehydrogenase (BDH) were additionally expressed to avoid clogging the carbon flux towards 2,3-BDO biosynthesis [15].

**Fig. S2 Prolonged fermentation profiles of some YHI030 strains in flask-scale batch cultivation using synthetic medium.**

Time-course data for (A) cell density (OD_600_) and (B) concentrations (mM) of glucose, glycerol, ethanol, and acetate. YHI030 strains were cultured in 50 mL of medium in a 200- mL baffled flask shaken at 120 rpm. Data are expressed as the mean ± SD (n = 3).

**Fig. S3 Effects of expression levels of opt_ecoFBPase, ATPase in BY318 and YHI030 strains.**

Specific rate of glucose consumption and ethanol production in BY318 strains and that in YHI030 strains during flask-scale batch cultivation using a synthetic medium. Each rate was determined from the exponential growth data. Error bars indicate standard deviation, and asterisks indicate the results of the two-sided *t*-test (**p* < 0.05, ***p* < 0.01, ****p* < 0.001, n = 3).

**Fig. S4 Comparison of adenylate energy charge of BY318 and YHI030 strains.**

Adenylate energy charge (EC) of BY318 and YHI030 strains expressing either opt_ecoFBPase or ATPase, in addition to the control strain. Error bars indicate the standard deviation, and asterisks indicate the results of a two-sided *t*-test (**p* < 0.05, ***p* < 0.01, ****p* < 0.001, n = 3).

**Table S1 Metabolite concentration in BY318 strains normalized to d-camphor sulfonic acid (set = 1)**

| **Metabolites** | **BY318-Control** | | | **BY318-opt_ecoFBPase** | | | **BY318-ATPase** | | |
| --- | --- | --- | --- | --- | --- | --- | --- | --- | --- |
| Lys | 0.39696 | 0.42511 | 0.45127 | 0.70625 | 0.71244 | 0.61112 | 0.52319 | 0.54147 | 0.55516 |
| Arg | 0.13697 | 0.17522 | 0.17495 | 0.12676 | 0.11553 | 0.14247 | 0.14978 | 0.16676 | 0.15337 |
| His | 0.03991 | 0.06236 | 0.04830 | 0.04636 | 0.02481 | 0.05285 | 0.02918 | 0.02861 | 0.03369 |
| Gly | 0.01044 | 0.01043 | 0.01152 | 0.01145 | 0.01189 | 0.01221 | 0.01237 | 0.01207 | 0.01286 |
| Ser | 0.01247 | 0.01326 | 0.01446 | 0.01767 | 0.01956 | 0.01631 | 0.01928 | 0.01904 | 0.02067 |
| Asn | 0.02608 | 0.02650 | 0.02822 | 0.04599 | 0.05367 | 0.04046 | 0.04576 | 0.04446 | 0.04925 |
| Ala | 0.03058 | 0.03226 | 0.03423 | 0.05916 | 0.06208 | 0.04913 | 0.06688 | 0.06762 | 0.06939 |
| Gln | 0.15801 | 0.17271 | 0.17917 | 0.27874 | 0.28805 | 0.23749 | 0.20398 | 0.21065 | 0.21646 |
| Thr | 0.01729 | 0.01858 | 0.02085 | 0.03050 | 0.03052 | 0.03036 | 0.03498 | 0.03776 | 0.03782 |
| Cys | 0.00000 | 0.00001 | 0.00000 | 0.00001 | 0.00000 | 0.00001 | 0.00001 | 0.00000 | 0.00000 |
| Met | 0.00059 | 0.00057 | 0.00058 | 0.00044 | 0.00052 | 0.00046 | 0.00070 | 0.00062 | 0.00076 |
| Tyr | 0.01319 | 0.01349 | 0.01436 | 0.00932 | 0.01191 | 0.00844 | 0.00941 | 0.00993 | 0.01021 |
| Glu | 0.63586 | 0.60646 | 0.60195 | 0.71564 | 0.78812 | 0.71739 | 0.67402 | 0.69733 | 0.68868 |
| Asp | 0.09049 | 0.07683 | 0.07056 | 0.10623 | 0.12063 | 0.13298 | 0.11004 | 0.11440 | 0.11868 |
| Phe | 0.00541 | 0.00554 | 0.00575 | 0.00570 | 0.00762 | 0.00504 | 0.01075 | 0.01144 | 0.01173 |
| 2KG | 0.03008 | 0.03311 | 0.03313 | 0.03008 | 0.02131 | 0.02987 | 0.02141 | 0.02273 | 0.02196 |
| Trp | 0.00691 | 0.00701 | 0.00791 | 0.00518 | 0.00487 | 0.00386 | 0.00482 | 0.00359 | 0.00499 |
| G3P | 0.01344 | 0.01047 | 0.01220 | 0.00250 | 0.00391 | 0.00225 | 0.00261 | 0.00196 | 0.00213 |
| G1P | 0.00205 | 0.00170 | 0.00191 | 0.00259 | 0.00363 | 0.00206 | 0.00238 | 0.00218 | 0.00188 |
| G6P | 0.02498 | 0.02070 | 0.02070 | 0.04595 | 0.02372 | 0.04499 | 0.01669 | 0.01429 | 0.01191 |
| R5P | 0.00233 | 0.00212 | 0.00363 | 0.00185 | 0.00286 | 0.00170 | 0.00303 | 0.00294 | 0.00243 |
| S7P | 0.01435 | 0.01268 | 0.01261 | 0.01362 | 0.01012 | 0.01573 | 0.00640 | 0.00367 | 0.00535 |
| F6P | 0.00848 | 0.00746 | 0.00752 | 0.01687 | 0.00912 | 0.01256 | 0.00587 | 0.00548 | 0.00420 |
| FBP | 0.01948 | 0.04087 | 0.04678 | 0.02462 | 0.01656 | 0.06805 | 0.00505 | 0.00364 | 0.00556 |
| DHAP | 0.00344 | 0.00834 | 0.00667 | 0.00597 | 0.00162 | 0.00755 | 0.00196 | 0.00219 | 0.00184 |
| GAP | 0.00049 | 0.00062 | 0.00031 | 0.00031 | 0.00023 | 0.00058 | 0.00033 | 0.00015 | 0.00029 |
| 3PG+2PG | 0.07280 | 0.04912 | 0.03487 | 0.01991 | 0.02120 | 0.01885 | 0.01676 | 0.01489 | 0.01502 |
| PEP | 0.01340 | 0.01102 | 0.00767 | 0.00459 | 0.00590 | 0.00290 | 0.00625 | 0.00409 | 0.00536 |
| Pyr | 0.00086 | 0.00091 | 0.00078 | 0.00093 | 0.00066 | 0.00059 | 0.00088 | 0.00072 | 0.00092 |
| 6PG | 0.00483 | 0.00485 | 0.00289 | 0.00310 | 0.00209 | 0.00304 | 0.00231 | 0.00097 | 0.00241 |
| Xu5P | 0.00370 | 0.00553 | 0.00449 | 0.00576 | 0.00144 | 0.00647 | 0.00295 | 0.00174 | 0.00277 |
| cAMP | 0.00025 | 0.00019 | 0.00017 | 0.00035 | 0.00059 | 0.00046 | 0.00039 | 0.00049 | 0.00045 |
| AMP | 0.03345 | 0.03357 | 0.01909 | 0.05187 | 0.05600 | 0.03667 | 0.20584 | 0.21384 | 0.22561 |
| ADP | 0.09987 | 0.08986 | 0.06134 | 0.06833 | 0.10762 | 0.05685 | 0.09473 | 0.09601 | 0.10007 |
| ATP | 0.63644 | 0.72201 | 0.67656 | 0.83978 | 0.78032 | 0.87482 | 0.35910 | 0.39209 | 0.36486 |
| cGMP | 0.00000 | 0.00006 | 0.00001 | 0.00005 | 0.00007 | 0.00008 | 0.00001 | 0.00002 | 0.00001 |
| GMP | 0.00140 | 0.00111 | 0.00059 | 0.00059 | 0.00106 | 0.00042 | 0.01821 | 0.01917 | 0.01941 |
| GDP | 0.01145 | 0.01206 | 0.00946 | 0.01110 | 0.01311 | 0.00975 | 0.01336 | 0.01442 | 0.01388 |
| GTP | 0.06020 | 0.06585 | 0.05224 | 0.07595 | 0.08680 | 0.07295 | 0.04259 | 0.03877 | 0.04132 |
| TMP | 0.00092 | 0.00054 | 0.00049 | 0.00040 | 0.00058 | 0.00020 | 0.00244 | 0.00238 | 0.00270 |
| TDP | 0.00089 | 0.00069 | 0.00045 | 0.00044 | 0.00083 | 0.00029 | 0.00058 | 0.00053 | 0.00060 |
| TTP | 0.01416 | 0.01683 | 0.01675 | 0.01677 | 0.01672 | 0.01688 | 0.00612 | 0.00629 | 0.00571 |
| CMP | 0.00308 | 0.00253 | 0.00142 | 0.00150 | 0.00247 | 0.00111 | 0.02431 | 0.02627 | 0.02669 |
| CDP | 0.00590 | 0.00486 | 0.00307 | 0.00269 | 0.00521 | 0.00232 | 0.00465 | 0.00409 | 0.00513 |
| CTP | 0.04946 | 0.05539 | 0.05087 | 0.04895 | 0.04902 | 0.05523 | 0.02856 | 0.03353 | 0.02861 |
| UMP | 0.00028 | 0.00022 | 0.00015 | 0.00000 | 0.00016 | 0.00006 | 0.00207 | 0.00204 | 0.00214 |
| UDP | 0.01165 | 0.01173 | 0.00590 | 0.00670 | 0.00902 | 0.00598 | 0.03030 | 0.02753 | 0.02510 |
| UTP | 0.18273 | 0.26385 | 0.21622 | 0.24318 | 0.22991 | 0.26886 | 0.22799 | 0.26992 | 0.23504 |
| Orotate | 0.03722 | 0.04023 | 0.02570 | 0.11465 | 0.15091 | 0.10730 | 0.80911 | 0.84567 | 0.87537 |
| NAD | 0.28620 | 0.30417 | 0.30449 | 0.33182 | 0.36326 | 0.28185 | 0.43087 | 0.45870 | 0.44687 |
| NADH | 0.00475 | 0.00839 | 0.00870 | 0.00175 | 0.00120 | 0.00270 | 0.00105 | 0.00150 | 0.00114 |
| NADP | 0.01644 | 0.03018 | 0.02340 | 0.02169 | 0.01444 | 0.02496 | 0.01528 | 0.01153 | 0.01615 |
| NADPH | 0.00337 | 0.00369 | 0.00336 | 0.00198 | 0.00153 | 0.00159 | 0.00130 | 0.00148 | 0.00100 |
| SuCoA | 0.00004 | 0.00004 | 0.00000 | 0.00000 | 0.00000 | 0.00000 | 0.00000 | 0.00002 | 0.00005 |
| AcCoA | 0.02520 | 0.02596 | 0.02219 | 0.03242 | 0.01250 | 0.03525 | 0.01475 | 0.01963 | 0.02131 |
| Carbamoyl phosphate | 0.00000 | 0.00001 | 0.00001 | 0.00000 | 0.00000 | 0.00000 | 0.00000 | 0.00001 | 0.00000 |
| PRPP | 0.00625 | 0.00296 | 0.00199 | 0.00724 | 0.01075 | 0.00624 | 0.00525 | 0.00120 | 0.00322 |
| cis-Aconitate | 0.00668 | 0.00616 | 0.00432 | 0.01002 | 0.01333 | 0.01132 | 0.00818 | 0.00880 | 0.00911 |
| Succinate | 0.12521 | 0.14536 | 0.14759 | 0.17320 | 0.20674 | 0.17159 | 0.34598 | 0.39535 | 0.38766 |
| Malate | 0.05020 | 0.04730 | 0.03655 | 0.06848 | 0.05017 | 0.03342 | 0.06507 | 0.04650 | 0.06500 |
| Fumarate | 0.00051 | 0.00187 | 0.00218 | 0.00218 | 0.00086 | 0.00222 | 0.00115 | 0.00091 | 0.00115 |
| CoA-SH | 0.00663 | 0.00582 | 0.00286 | 0.00224 | 0.00113 | 0.00223 | 0.00089 | 0.00120 | 0.00188 |
| Trehalose | 0.14327 | 0.16416 | 0.17775 | 0.16556 | 0.16340 | 0.17098 | 0.09515 | 0.10352 | 0.10388 |
| UDP-Glc | 0.23334 | 0.25497 | 0.21485 | 0.55244 | 0.71021 | 0.38806 | 0.99152 | 1.06223 | 1.00477 |
| T6P | 0.00652 | 0.00681 | 0.00606 | 0.00354 | 0.00638 | 0.00351 | 0.00252 | 0.00248 | 0.00317 |
| Citric acid | 1.07028 | 1.25149 | 0.83586 | 1.82135 | 1.26390 | 2.04102 | 1.11018 | 1.15821 | 1.29826 |

**Table S2 Metabolite concentration in YHI030 strains normalized to d-camphor sulfonic acid (set = 1)**

| **Metabolites** | **YHI030-Control** | | | **YHI030-opt_ecoFBPase** | | | **YHI030-ATPase** | | |
| --- | --- | --- | --- | --- | --- | --- | --- | --- | --- |
| Lys | 0.61479 | 0.38667 | 0.46554 | 0.41731 | 0.41663 | 0.45917 | 0.42122 | 0.39150 | 0.38322 |
| Arg | 0.17958 | 0.01728 | 0.09889 | 0.02734 | 0.02773 | 0.07347 | 0.02998 | 0.01991 | 0.02559 |
| His | 0.39099 | 0.08940 | 0.30331 | 0.08776 | 0.09103 | 0.24862 | 0.09111 | 0.07424 | 0.10047 |
| Gly | 0.00586 | 0.00375 | 0.00572 | 0.00444 | 0.00422 | 0.00563 | 0.00494 | 0.00392 | 0.00457 |
| Ser | 0.01551 | 0.01468 | 0.01496 | 0.01304 | 0.01207 | 0.01350 | 0.01407 | 0.01377 | 0.01511 |
| Asn | 0.02114 | 0.01892 | 0.01960 | 0.02343 | 0.02413 | 0.01951 | 0.02515 | 0.02049 | 0.01985 |
| Ala | 0.18543 | 0.05176 | 0.10368 | 0.06442 | 0.06287 | 0.08637 | 0.07656 | 0.05986 | 0.05924 |
| Gln | 0.26439 | 0.15361 | 0.19466 | 0.16443 | 0.16502 | 0.19256 | 0.16658 | 0.15385 | 0.15113 |
| Thr | 0.02773 | 0.01544 | 0.02475 | 0.01633 | 0.01602 | 0.02069 | 0.01877 | 0.01540 | 0.01261 |
| Cys | 0.00000 | 0.00000 | 0.00000 | 0.00000 | 0.00000 | 0.00000 | 0.00000 | 0.00000 | 0.00000 |
| Met | 0.00020 | 0.00057 | 0.00046 | 0.00065 | 0.00073 | 0.00040 | 0.00067 | 0.00054 | 0.00059 |
| Tyr | 0.00647 | 0.01448 | 0.00860 | 0.01236 | 0.01369 | 0.00726 | 0.00914 | 0.00878 | 0.00841 |
| Glu | 0.78010 | 0.34786 | 0.64287 | 0.45918 | 0.45828 | 0.52827 | 0.47444 | 0.40323 | 0.40120 |
| Asp | 0.02466 | 0.03663 | 0.02759 | 0.03279 | 0.02749 | 0.01604 | 0.03644 | 0.03679 | 0.04062 |
| Phe | 0.00409 | 0.00887 | 0.00491 | 0.00753 | 0.00827 | 0.00441 | 0.00578 | 0.00550 | 0.00540 |
| 2KG | 0.61772 | 0.11616 | 0.14169 | 0.07633 | 0.07116 | 0.10904 | 0.07621 | 0.08735 | 0.07271 |
| Trp | 0.03433 | 0.08560 | 0.06498 | 0.08550 | 0.07903 | 0.05117 | 0.06696 | 0.06616 | 0.06058 |
| G3P | 0.04812 | 0.04208 | 0.03543 | 0.06140 | 0.05371 | 0.01992 | 0.07279 | 0.05920 | 0.06194 |
| G1P | 0.00360 | 0.00617 | 0.00539 | 0.00971 | 0.00966 | 0.00513 | 0.01010 | 0.00751 | 0.00769 |
| G6P | 0.12206 | 0.14641 | 0.16365 | 0.24910 | 0.28607 | 0.15518 | 0.24595 | 0.18785 | 0.21826 |
| R5P | 0.03182 | 0.03037 | 0.01992 | 0.02605 | 0.01981 | 0.01444 | 0.02291 | 0.03105 | 0.01533 |
| S7P | 0.12741 | 0.19746 | 0.08362 | 0.10494 | 0.08612 | 0.04311 | 0.11529 | 0.13920 | 0.13607 |
| F6P | 0.03203 | 0.04446 | 0.04148 | 0.10830 | 0.09865 | 0.04542 | 0.10492 | 0.07693 | 0.05138 |
| FBP | 0.24268 | 0.08688 | 0.32525 | 0.24004 | 0.28295 | 0.57797 | 0.21446 | 0.14801 | 0.09910 |
| DHAP | 0.02947 | 0.02236 | 0.02070 | 0.04025 | 0.03790 | 0.02124 | 0.04076 | 0.02924 | 0.00896 |
| GAP | 0.00000 | 0.00039 | 0.00039 | 0.00076 | 0.00078 | 0.00069 | 0.00080 | 0.00050 | 0.00017 |
| 3PG+2PG | 0.05821 | 0.03287 | 0.02644 | 0.02144 | 0.01922 | 0.01511 | 0.02530 | 0.03005 | 0.02023 |
| PEP | 0.01046 | 0.00609 | 0.00315 | 0.00381 | 0.00263 | 0.00138 | 0.00370 | 0.00614 | 0.00461 |
| Pyr | 0.01026 | 0.00999 | 0.00698 | 0.00718 | 0.00565 | 0.00325 | 0.00790 | 0.00870 | 0.00736 |
| 6PG | 0.09700 | 0.06639 | 0.05590 | 0.02530 | 0.02307 | 0.01851 | 0.02639 | 0.02992 | 0.02732 |
| Xu5P | 0.06462 | 0.07053 | 0.04219 | 0.05122 | 0.04495 | 0.02165 | 0.05560 | 0.05377 | 0.02407 |
| cAMP | 0.00041 | 0.00022 | 0.00014 | 0.00021 | 0.00021 | 0.00028 | 0.00023 | 0.00023 | 0.00025 |
| AMP | 0.10785 | 0.01027 | 0.01942 | 0.01168 | 0.01480 | 0.02649 | 0.01056 | 0.00986 | 0.01768 |
| ADP | 0.36498 | 0.04063 | 0.07406 | 0.03857 | 0.04629 | 0.05201 | 0.04058 | 0.03946 | 0.05243 |
| ATP | 1.08653 | 0.49235 | 0.55111 | 0.51034 | 0.47660 | 0.37701 | 0.53743 | 0.47417 | 0.39725 |
| cGMP | 0.00002 | 0.00000 | 0.00000 | 0.00000 | 0.00000 | 0.00000 | 0.00000 | 0.00000 | 0.00000 |
| GMP | 0.00389 | 0.00063 | 0.00084 | 0.00052 | 0.00049 | 0.00054 | 0.00064 | 0.00067 | 0.00082 |
| GDP | 0.07205 | 0.00623 | 0.01101 | 0.00565 | 0.00668 | 0.00687 | 0.00699 | 0.00666 | 0.00604 |
| GTP | 0.15827 | 0.07469 | 0.06640 | 0.07881 | 0.07523 | 0.04475 | 0.08758 | 0.08628 | 0.05983 |
| TMP | 0.00913 | 0.00020 | 0.00061 | 0.00008 | 0.00011 | 0.00006 | 0.00015 | 0.00010 | 0.00010 |
| TDP | 0.00205 | 0.00019 | 0.00047 | 0.00016 | 0.00022 | 0.00016 | 0.00014 | 0.00016 | 0.00031 |
| TTP | 0.00511 | 0.00796 | 0.00361 | 0.00820 | 0.00852 | 0.00294 | 0.00822 | 0.00726 | 0.00508 |
| CMP | 0.00144 | 0.00048 | 0.00049 | 0.00078 | 0.00074 | 0.00075 | 0.00079 | 0.00067 | 0.00078 |
| CDP | 0.00807 | 0.00083 | 0.00160 | 0.00096 | 0.00121 | 0.00194 | 0.00105 | 0.00081 | 0.00149 |
| CTP | 0.04407 | 0.02973 | 0.02795 | 0.03361 | 0.03425 | 0.02362 | 0.03342 | 0.03078 | 0.02423 |
| UMP | 0.00000 | 0.00004 | 0.00000 | 0.00004 | 0.00006 | 0.00011 | 0.00005 | 0.00005 | 0.00008 |
| UDP | 0.03494 | 0.00318 | 0.00744 | 0.00382 | 0.00670 | 0.00889 | 0.00384 | 0.00476 | 0.00914 |
| UTP | 0.11965 | 0.16287 | 0.11209 | 0.22428 | 0.21646 | 0.08299 | 0.23347 | 0.20903 | 0.16202 |
| Orotate | 0.06562 | 0.31607 | 0.26908 | 0.15752 | 0.20695 | 0.06946 | 0.01846 | 0.00736 | 0.00725 |
| NAD | 0.40236 | 0.39988 | 0.40814 | 0.48734 | 0.47044 | 0.40116 | 0.45536 | 0.43706 | 0.41579 |
| NADH | 0.01764 | 0.00828 | 0.01726 | 0.01314 | 0.01122 | 0.01722 | 0.01426 | 0.01088 | 0.00753 |
| NADP | 0.08313 | 0.02552 | 0.04695 | 0.01571 | 0.01631 | 0.02669 | 0.01862 | 0.02381 | 0.01008 |
| NADPH | 0.01445 | 0.00408 | 0.00971 | 0.00304 | 0.00239 | 0.00560 | 0.00315 | 0.00343 | 0.00168 |
| SuCoA | 0.00034 | 0.00031 | 0.00025 | 0.00036 | 0.00075 | 0.00073 | 0.00029 | 0.00009 | 0.00029 |
| AcCoA | 0.04041 | 0.03504 | 0.07170 | 0.03716 | 0.03816 | 0.06399 | 0.03728 | 0.04180 | 0.02971 |
| Carbamoyl phosphate | 0.00004 | 0.00004 | 0.00010 | 0.00002 | 0.00003 | 0.00002 | 0.00000 | 0.00002 | 0.00000 |
| PRPP | 0.00384 | 0.00406 | 0.00691 | 0.00539 | 0.00478 | 0.00613 | 0.00623 | 0.00562 | 0.00109 |
| cis-Aconitate | 0.03946 | 0.00870 | 0.01008 | 0.01060 | 0.00834 | 0.00625 | 0.01140 | 0.00857 | 0.01097 |
| Succinate | 2.30195 | 0.23165 | 0.52378 | 0.32779 | 0.37590 | 0.48559 | 0.25726 | 0.21824 | 0.21100 |
| Malate | 2.49093 | 1.15123 | 1.59080 | 1.16809 | 1.19450 | 1.09404 | 1.20560 | 1.12600 | 1.16317 |
| Fumarate | 0.08313 | 0.03857 | 0.04008 | 0.03886 | 0.03813 | 0.02275 | 0.04311 | 0.03640 | 0.02595 |
| CoA-SH | 0.04211 | 0.00321 | 0.00905 | 0.00155 | 0.00459 | 0.00981 | 0.00144 | 0.00075 | 0.00270 |
| Trehalose | 0.21052 | 0.08588 | 0.14898 | 0.11352 | 0.10874 | 0.13766 | 0.12438 | 0.10386 | 0.10194 |
| UDP-Glc | 0.57040 | 0.40248 | 0.35929 | 0.57933 | 0.54889 | 0.35200 | 0.55808 | 0.49785 | 0.52423 |
| T6P | 0.01638 | 0.01629 | 0.01009 | 0.01142 | 0.01088 | 0.00605 | 0.01077 | 0.00736 | 0.00823 |
| Citric acid | 13.52270 | 0.94903 | 1.68206 | 1.05187 | 0.97980 | 1.20585 | 1.15748 | 0.83279 | 0.46757 |

**Table S3 Plasmids used in this study**

| **Plasmid** | **Description** | **References** |
| --- | --- | --- |
| pATP422-alsLpOp-aldcLlOp | pATP422, expressions of *L. plantarum* codon-optimized *als* (*alsLpOp*) gene by *PGK1* promoter and *L. lactis* codon-optimized ALDC (*aldcLlOp*) gene by *TDH3* promoter | [15] |
| pAT425-BDH1 | pAT425, expression of *S. cerevisiae*BDH (*BDH1*) gene by *ADH1* promoter | [15] |
| pGK426 | Yeast multi-copy type single-gene expression vector containing *PGK1* promoter, *PGK1* terminator, *2μ* origin, and *URA3* marker | [43] |
| pGEM-T-easy-sceFBPase | pGEM-T-easy, cloning of *S. cerevisiae* sceFBPase (*FBP1*) gene | This study |
| pGEM-T-easy-ecoFBPase | pGEM-T-easy, cloning of *E. coli* ecoFBPase (*fbp*) gene | This study |
| pGK426-sceFBPase | pGK426, expression of *S. cerevisiae* sceFBPase (*FBP1*) gene by *PGK1* promoter | This study |
| pGK426-ecoFBPase | pGK426, expression of *E. coli* ecoFBPase (*fbp*) gene by *PGK1* promoter | This study |
| pGK426-opt_ecoFBPase | pGK426, expression of *E. coli* codon-optimized opt_ecoFBPase (*fbp*) gene by *PGK1* promoter | This study |
| pGK426-atpAGD | pGK426, expression of *E. coli* ATPase α, β and γ subunit genes (atpAGD) joined with viral 2A peptide sequences gene by *PGK1* promoter | This study |

**Table S4 Primers used in this study**

| **Primer** | **Sequence (5′ to 3′)** |
| --- | --- |
| NheI_fbpSc_fw | GGCCGCTAGCATGCCAACTCTAGTAAATG |
| BglII_fbpSc_rv | GGGAGATCTCTACTGTGACTTGCCAATATGGTC |
| NheI_fbpEc_fw | AATTGCTAGCATGAAAACGTTAGGTGAATTTATTGTCG |
| BglII_fbpEc_rv | AATTAGATCTTTACGCGTCCGGGAACTCACG |
| pGK426_BglII_fw | AGATCTGAAATAAATTGAATTGAATTGAAATCGATAGATC |
| pGK426_NheI_rv | GCTAGCGTTTTATATTTGTTGTAAAAAGTAGATAATTACTTC |
| pGK426_NheI_atpA_fw | AACAAATATAAAACGCTAGCATGCAACTGAATTCCACCGAAATCAG |
| ERBV-1_atpA_rv | TCACCAGCCAATTTCAACAAAGAAAAATTAGTAGCACCTCCAGATCCCCAGGATTGGGTTGCTTTGAAGG |
| ERBV-1_atpG_fw | GTTGAAATTGGCTGGTGATGTTGAATTGAATCCAGGTCCAATGGCCGGCGCAAAAGA |
| P2A_atpG_rv | CACCAGCTTGTTTCAACAAAGAAAAATTAGTAGCTCCAGATCCAACCGCGGCGGCCC |
| P2A_atpD_fw | TGAAACAAGCTGGTGATGTTGAAGAAAATCCAGGTCCAATGGCTACTGGAAAGATTGTCCAG |
| pGK426_BglII_atpD_rv | ATTCAATTTATTTCAGATCTTTAAAGTTTTTTGGCTTTTTCCACAGCTTC |
| atpA_sq1 | CGTCCACCATTTCTAACGT |
| atpA_sq2 | GTATGCGCCGATGTCCGTTG |
| atpG_sq3 | GCCCAGGTCACCGGC |
| atpD_sq4 | ACGGTCTGCGTCGCGG |
| ACT1_for_RT-PCR_F | TTGGATTCCGGTGATGGTGTTAC |
| ACT1_for_RT-PCR_R | ACCACGTTCACTCAAGATCTTCA |
| ATPase_for_RT-PCR_F | GTGACGGTGTTATCCGCATTCAC |
| ATPase_for_RT-PCR_R | AGCGTACGGACCCATAACAAC |
| optFBPase_for_RT-PCR_F | AGGCTAGAGATATCGTTGCTGG |
| optFBPase_for_RT-PCR_R | GTTAGAGGAGCCATCCAAAGGATC |
